# Dual COX and 5-LOX inhibition by clerodane diterpenes from seeds of *Polyalthia longifolia* (Sonn.) Thwaites

**DOI:** 10.1038/s41598-020-72840-8

**Published:** 2020-09-29

**Authors:** Ha Thi Nguyen, Thien-Y. Vu, Vishala Chandi, Haritha Polimati, Vinay Bharadwaj Tatipamula

**Affiliations:** 1 Institute of Research and Development, Duy Tan University, Da Nang, 550000 Vietnam; 2 Faculty of Medicine, Duy Tan University, Da Nang, 550000 Vietnam; 3 Faculty of Pharmacy, Ton Duc Thang University, Ho Chi Minh City, 700000 Vietnam; 4 Pharmacology Department, AU College of Pharmaceutical Sciences, Andhra University, Visakhapatnam, Andhra Pradesh 530003 India; 5 Pharmacology and Toxicology Division, Incozen Therapeutics Pvt Ltd., Turkapally, Telangana 500078 India

**Keywords:** Chemical biology, Computational biology and bioinformatics, Chemistry

## Abstract

Natural metabolites with their specific bioactivities are being considered as a potential source of materials for pharmacological studies. In this study, we successfully isolated and identified five known clerodane diterpenes, namely 16-oxo-cleroda-3,13(14)E-dien-15-oic acid **(1)**, 16-hydroxy-cleroda-3,13-dien-15-oic acid **(2)**, 16-hydroxy-cleroda-4(18),13-dien-16,15-olide **(3)**, 3α,16α-dihydroxy-cleroda-4(18),13(14)Z-dien-15,16-olide **(4)**, and 16α-hydroxy-cleroda-3,13(14)Z-dien-15,16-olide **(5)** from the methanolic extract of seeds of *Polyalthia longifolia*. Initially, all the isolated metabolites were investigated for COX-1, COX-2, and 5-LOX inhibitory activities using the standard inhibitory kits. Of which, compounds **3**, **4**, and **5** exhibited to be potent COX-1, COX-2, and 5-LOX inhibitors with the IC_50_ values similar or lower to those of the reference drugs. To understand the underlying mechanism, these compounds were subjected to molecular docking on COX-1, COX-2, and 5-LOX proteins. Interestingly, the in silico study results were in high accordance with in vitro studies where compounds **3**, **4**, and **5** hits assumed interactions and binding pattern comparable to that of reference drugs (indomethacin and diclofenac), as a co-crystallized ligand explaining their remarkable dual (COX/LOX) inhibitor actions. Taken together, our findings demonstrated that compounds **3, 4,** and **5** functioned as dual inhibitors of COX/5-LOX and can contribute to the development of novel, more effective anti-inflammatory drugs with minimal side-effects.

## Introduction

Cyclooxygenases (COX-1/2) and lipoxygenases (LOX) are well-recognized as pro-inflammatory enzymes as well as vital enzymes in the metabolic pathway of arachidonic acid^1^. These enzymes are essential for the physiological production of eicosanoids, including leukotrienes, prostaglandins, and thromboxane, that primarily cause inflammation. Therefore, COX and LOX enzymes are the rate-limiting steps in the production of eicosanoids and commonly used for screening and evaluating anti-inflammatory drugs.

COX-1 is a fundamental enzyme that is localized mostly in the kidney, stomach, and platelets of the human body and helped to maintain the renal and gastric homeostasis^2^. COX-1 catalyzes the biosynthesis of eicosanoids propagating platelet aggregation, thromboxanes, and vasoconstriction^3,4^. Consequently, long-term use of selective COX-1 inhibitors such as aspirin in the treatment of some chronic inflammation may cause hemorrhage, kidney failure, and/or severe side effects on gastrointestinal^5–7^.

COX-2 is an inducible enzyme that presents in low amounts in the human kidney, brain, and ovaries^2^. Its expression is induced in the case of mitogenic stimulation, inflammation, or tissue damage^8^. Inhibition of COX-2 leads to reduced production of prostacyclin, a vasodilator, and anti-aggregatory prostanoid^9^. As a result, selective COX-2 inhibitors considerably minimize gastrointestinal side effects but increase the likelihood of cardiovascular diseases such as congestive heart failure, systemic and pulmonary hypertension, and myocardial infarction^6,10,11^.

5-lipoxygenase (5-LOX), on the other hand, is one isozyme of LOX, which activates the second essential biosynthetic pathway to liberate eicosanoids^12^. The 5-LOX pathway’s end product is leukotriene B4, a mediator of several inflammatory and allergic diseases, including atherosclerosis, cancer, and cardiovascular diseases^12–15^. Thus, reducing the leukotrienes via inhibiting 5-LOX may help to reduce the potential risk of cardiovascular and gastrointestinal disease caused by selective COX-2 and COX-1 inhibitors^16^, respectively.

It is noteworthy that COX/5-LOX co-inhibition potentially reduces side effects on the cardiovascular and gastrointestinal tract while retaining the primary activity of COX-1/2 inhibitors. With this aim, darbufelone and licofelone were first designed and clinically used as a dual COX/5-LOX inhibitory anti-inflammatory drugs. However, due to the high toxicity and/or limited efficacy, they were unable to be marketed^17,18^, making the development and bioactivity studies of dual inhibitors are still in urgent need. As it is noted that natural products and their semi-synthetic derivatives are potentially safer and more active than the synthetic compounds^19,20^, the metabolites from natural sources are extensively screened for their dual inhibitor properties. Several previous studies^21,22^ have demonstrated good in vivo anti-inflammatory activities of *Polyalthia longifolia* (Family: Annonaceae) extracts. However, the specific metabolites that are responsible for this activity and the proper enzymatic investigations against COX-1, COX-2, and 5-LOX remained undetermined. In the present study, we first isolated and identified the metabolites present in methanolic extracts of seeds of *P. longifolia*. Afterward, these metabolites were screened for their potential COX-1/2 and 5-LOX dual inhibitory activities by performing in vitro experiments. Their dual inhibitory activity and binding mode were then investigated in silico using Schrodinger suite 2020-2.

## Results

### Isolation and identification of the compounds

**Figure 1:**
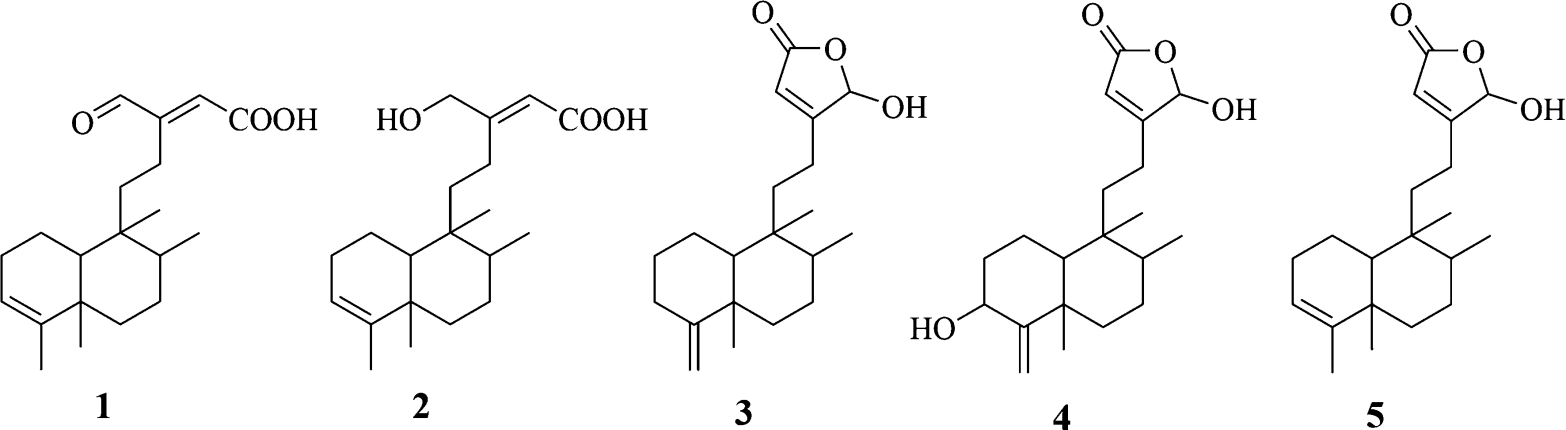
Known secondary metabolites isolated from methanolic extract of seeds of *Polyalthia longifolia* (Sonn.) Thwaites. 16-oxo-cleroda-3,13(14)E-dien-15-oic acid **(1)**, 16-hydroxy-cleroda-3,13-dien-15-oic acid **(2)**, 16-hydroxy-cleroda-4(18),13-dien-16,15-olide **(3)**, 3α,16α-dihydroxy-cleroda-4(18),13(14)Z-dien-15,16-olide **(4)**, and 16α-hydroxy-cleroda-3,13(14)Z-dien-15,16-olide **(5)**.

Five known compounds **(1–5)** were successfully isolated and identified from the methanolic extract of seeds of *P. longifolia* **(ME)** by utilizing chromatographic methods and analyses of their spectral data (Figures S1–S15). The obtained data were interrelated with those reported in the previous literature and the compounds were found to be 16-oxo-cleroda-3,13(14)E-dien-15-oic acid **(1)**^23^, 16-hydroxy-cleroda-3,13-dien-15-oic acid **(2)**^24^, 16-hydroxy-cleroda-4(18),13-dien-16,15-olide **(3)**^25^, 3α,16α-dihydroxy-cleroda-4(18),13(14)Z-dien-15,16-olide **(4)**^26^, and 16α-hydroxy-cleroda-3,13(14)Z-dien-15,16-olide **(5)**^23^ (Fig. 1). Although all of these compounds have been earlier reported from *P. longifolia*, it was the first time they were isolated from the seeds of this species.

### Anti-inflammatory effect of clerodane diterpenes (1–5) in vitro

**Figure 2:**
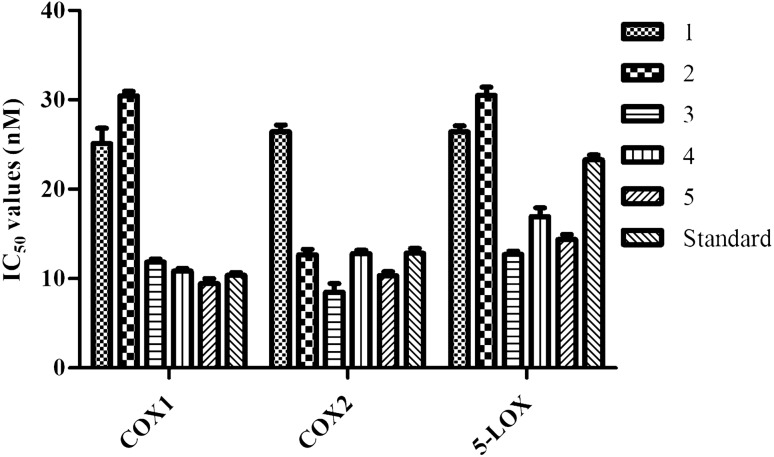
IC_50_ values of **1–5** against cyclooxygenase (COX-1 and COX-2) and 5-lipoxygenase (5-LOX) enzymes. Experiments were done three times (*n* = 3) and the data were presented as mean ± SD.

Based on the previous reports on the anti-inflammatory properties of the extracts of *P. longifolia,* we investigated the anti-inflammatory activities of these compounds in vitro. The anti-inflammatory effect of clerodane diterpenes* (1–5)* was assessed based on their ability to inhibit COX-1/2, and 5-LOX enzymes (Tables S1, S2). Our COX-1 inhibition results showed that only compound **5** had a higher percentage of inhibition (92.94 ± 2.46%) as compared to the reference drug indomethacin (85.87 ± 1.42%) at 10.0 µg/mL (Table S1). In addition, the concentration of compound **5** needed to inhibit COX-1 activity by 50% was 9.46 ± 0.33 nM, which was also noted to be better than indomethacin (10.39 ± 0.16 nM). The COX-1 inhibition effect of compounds **3** and **4** exhibited to be almost equivalent to that of the indomethacin with an IC_50_ value of 11.85 ± 0.19 and 10.85 ± 0.17 nM, respectively. While IC_50_ values of two remained, compounds **1** and **2** were 25.11 ± 0.99 and 30.44 ± 0.30, respectively (Fig. 2).

Concerning COX-2 inhibition capacity, the percentage of inhibition of compounds **2, 3** and **5** showed to be 84.98 ± 1.17, 82.97 ± 0.44, and 79.41 ± 4.39%, respectively, which were higher than the indomethacin (75.74 ± 3.21%). Compounds **4** showed similar inhibition activity as compared to indomethacin with an inhibition rate of 75.14 ± 2.73%. And only compound **1** exposed lower inhibition capacity at the rate of 62.85 ± 3.84% (Table S1). Similarly, compounds **3, 5, 2,** and **4** also showed to be better anti-inflammatory inhibitors than indomethacin (IC_50_ = 12.84 ± 0.32 nM) with the IC_50_ values of 8.49 ± 0.55, 10.34 ± 0.26, 12.70 ± 0.34, and 12.82 ± 0.21 nM, respectively, and that of compound **1** was 26.42 ± 0.45 nM (Fig. 2).

In accordance with the COX-1 and COX-2 assays, our results also displayed that except for compounds **1** and **2**, all other compounds (**3**–**5**) revealed a prominent percentage of inhibition against 5-LOX (Table S2). Particularly, the compounds **3, 5,** and** 4** showed better inhibition of 5-LOX enzyme than the standard drug diclofenac (23.28 ± 0.31 nM) with the concentration required for 50% inhibition of 5-LOX were found to be 12.73 ± 0.21, 14.38 ± 0.32, and 16.94 ± 0.56 nM, respectively. While this value of compound **1** and** 2** were 26.41 ± 0.39 and 30.51 ± 0.53 nM, respectively (Fig. 2).

### Docking studies

In order to confirm the possible binding of five isolated metabolites **(1–5)** with pro-inflammatory receptors, namely COX-1 (PDB ID: 2OYU), COX-2 (PDB ID: 4COX), and 5-LOX (PDB ID: 3V99), using Schrödinger software 2020-2 (Figures S16–S17). The positions at which the isolated metabolites formed binding residues and hydrogen bonds were investigated (Figure S18). The metal coordination presentations were analyzed using UCSF Chimera 1.14, and the scatter plots were designed by Tableau 2020.2. The Standard-precision (SP) and Extra-precision (XP) values of docking scores were reserved only for the criteria of reliable and correct binding mode but not for the free binding energy estimation.

**Figure 3:**
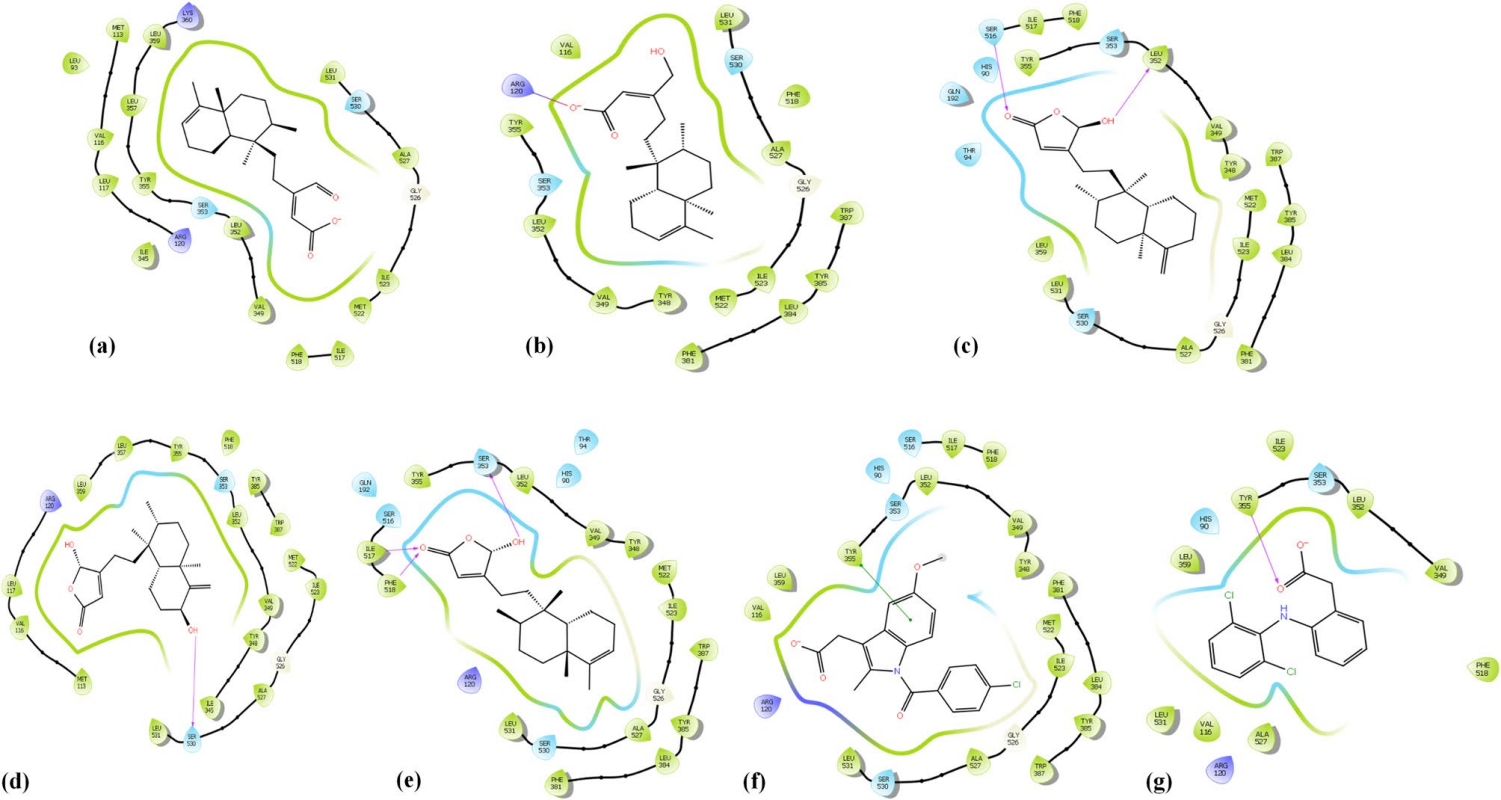
(**a**) Docking pose of compound **1**, (**b**) docking pose of compound **2**, (**c**) docking pose of compound **3**, (**d**) docking pose of compound **4**, (**e**) docking pose of compound **5**, (**f**) docking pose of indomethacin, (**g**) docking pose of diclofenac ligands—with COX-1 (PDB ID: 2OYU) protein.

**Figure 4:**
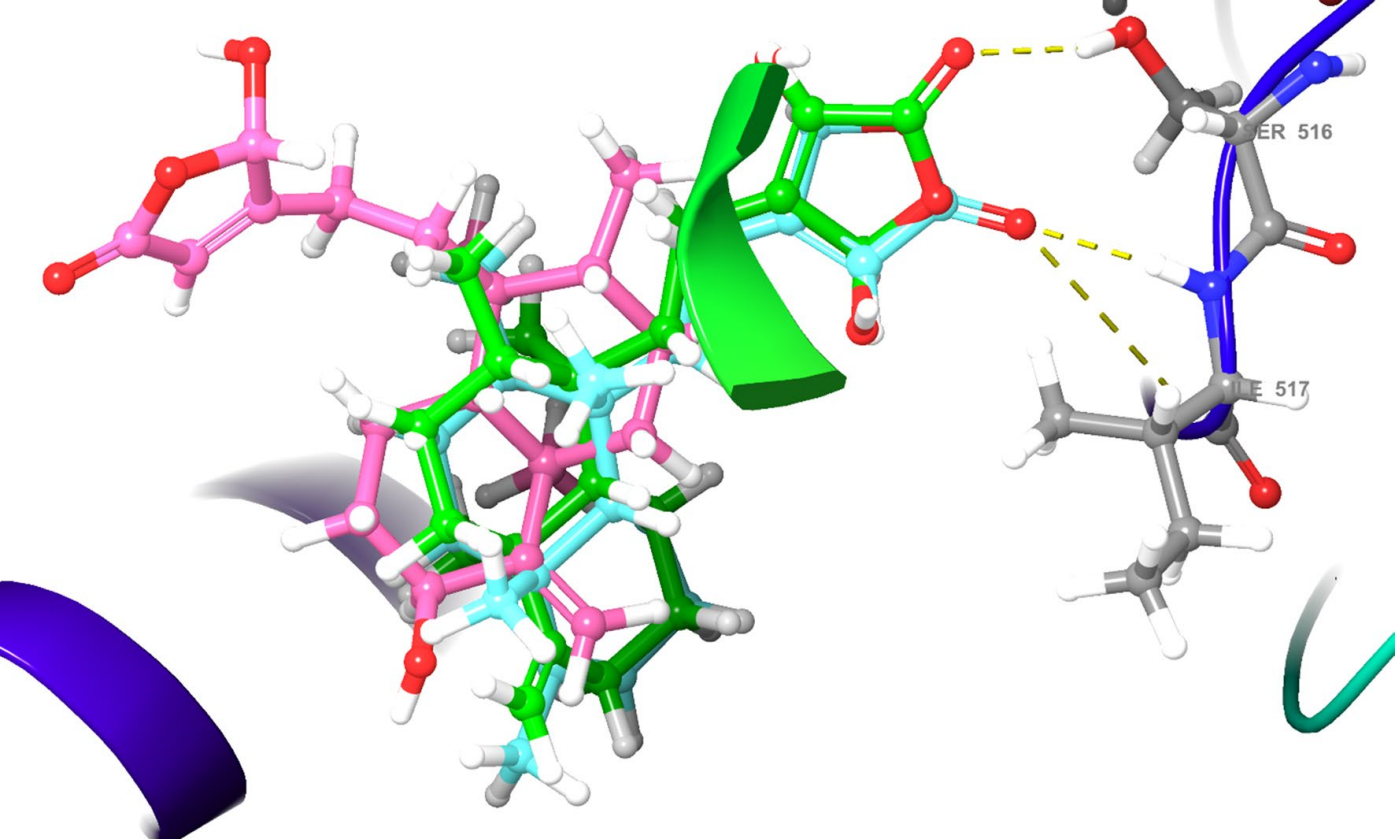
The visual inspection of ligand **3** (green), **4** (pink), and **5** (cyan) in COX-1 protein. Ligand **3** and **5** form H-bonds with “gate-holder” Ser 516, Leu 352 or Ile 517, Phe 518, Ser 353 residues while ligand **4** positioned interior and penetrated more deeply than ligands **3** and **5**.

The Root-Mean-Square deviation (RMSD) of native ligand at 0.64 Å after redocking to COX-1 protein showed a good binding mode of Glide programs. Of which, compound **4** and **5** prominently inhibited COX-1 with ΔG_MM-GBSA_ prediction values of − 57.7 and − 55.3 kcal/mol, respectively, which were found to be better inhibitors as compared to the reference drug indomethacin (− 47.5 kcal/mol) (Table 1). Metabolites **1, 2,** and **3**, on the other hand, presented lower COX-1 inhibition with ΔG_MM-GBSA_ prediction values of − 9.9, 5.6, and − 15.0 kcal/mol, respectively (Table 1). Structurally, there was a relative correlation between the number of H-bonds and the ΔG_MM-GBSA_ value. Compound **5** has three H-bonds with Ile517, Phe518, and Ser353; compound **3** has two H-bonds with Ser516 and Leu352; compounds **2** and **4** have only one H-bond with Arg120 and Ser530, respectively; and compound** 1** has none of H-bonds (Fig. 3; Table 2). The presence of polar carboxylate, aldehyde, or hydroxyl group in the hydrophobic pocket had generated the unfavorable hydrophobic interactions of compounds **1** and **2,** making their ΔG_MM-GBSA_ estimations decreased sharply. In compounds **3** and **5**, on the other hand, these groups were supported by polar residues His90, Thr94, Gln192, Ser353, and Ser516 that end-up with better ΔG_MM-GBSA_ scores (Table 2). The visual inspection of compound **4** (pink) in COX-1 protein showed its binding pose in the upper half of the active site (Fig. 4). This direction created fewer H-bonds and favorable hydrophobic interactions as compared to compounds **3** and **5** and made the compound penetrate more deeply in COX-1 protein. These findings could partly be explained for the high ΔG_MM-GBSA_ prediction and COX-1 inhibitor capacity of compound **4.**

**Table 1:** The XP docking scores, MM-GBSA estimations and experimental free energies (kcal/mol) of compounds **1–5** and reference drugs (indomethacin, and diclofenac) with COX-1, COX-2, and 5-LOX proteins.

Compounds	COX-1			COX-2			5-LOX		
	GlideScore (kcal/mol)	Free energy (kcal/mol)		GlideScore (kcal/mol)	Free energy (kcal/mol)		GlideScore (kcal/mol)	Free energy (kcal/mol)	
	XP	MM-GBSA	exp	XP	MM-GBSA	exp	XP	MM-GBSA	exp
**1**	− 7.3	− 9.9	− 6.3	− 9.3	− 30.6	− 6.5	− 7.8	− 18.4	− 6.2
**2**	− 8.0	5.6	− 6.2	− 9.4	− 15.1	− 6.4	− 7.1	− 34.0	− 6.3
**3**	− 10.1	− 15.0	− 6.8	− 10.2	− 36.8	− 6.9	− 5.2	− 42.1	− 6.5
**4**	− 10.1	− 57.7	− 6.9	− 9.6	− 39.2	− 7.0	− 6.1	− 35.1	− 6.3
**5**	− 10.2	− 55.3	− 6.7	− 9.9	− 38.9	− 6.9	− 5.3	− 50.0	− 6.5
**Indomethacin**	− 9.6	− 47.5	− 6.8	− 4.0	− 29.1	− 6.9	− 5.7	− 45.0	ND
**Diclofenac**	− 6.2	− 12.6	ND	− 8.9	− 49.6	ND	− 7.3	− 26.6	− 6.3

*ND* Not done.

**Table 2:** H-bond interactions in COX-1, COX-2, and 5-LOX proteins.

Compounds	COX-1		COX-2		5-LOX	
	No of H-bonds	Residues	No of H-bonds	Residues	No of H-bonds	Residues
**1**	0	–	2	Arg120, Tyr355	0	–
**2**	1	Arg120	2	Arg120, Tyr355	1	Ala672
**3**	2	Ser516, Leu352	1	Tyr355	1	Val175
**4**	1	Ser530	2	Arg120, Tyr355	2	Asp176, Val671
**5**	3	Ile517, Phe518, Ser353	1	Tyr355	1	Asn554
**Indomethacin**	1	Tyr355	1	Arg120	1	Val175
**Diclofenac**	1	Tyr355	2	Tyr355, Arg120	1	Lys173

**Figure 5:**
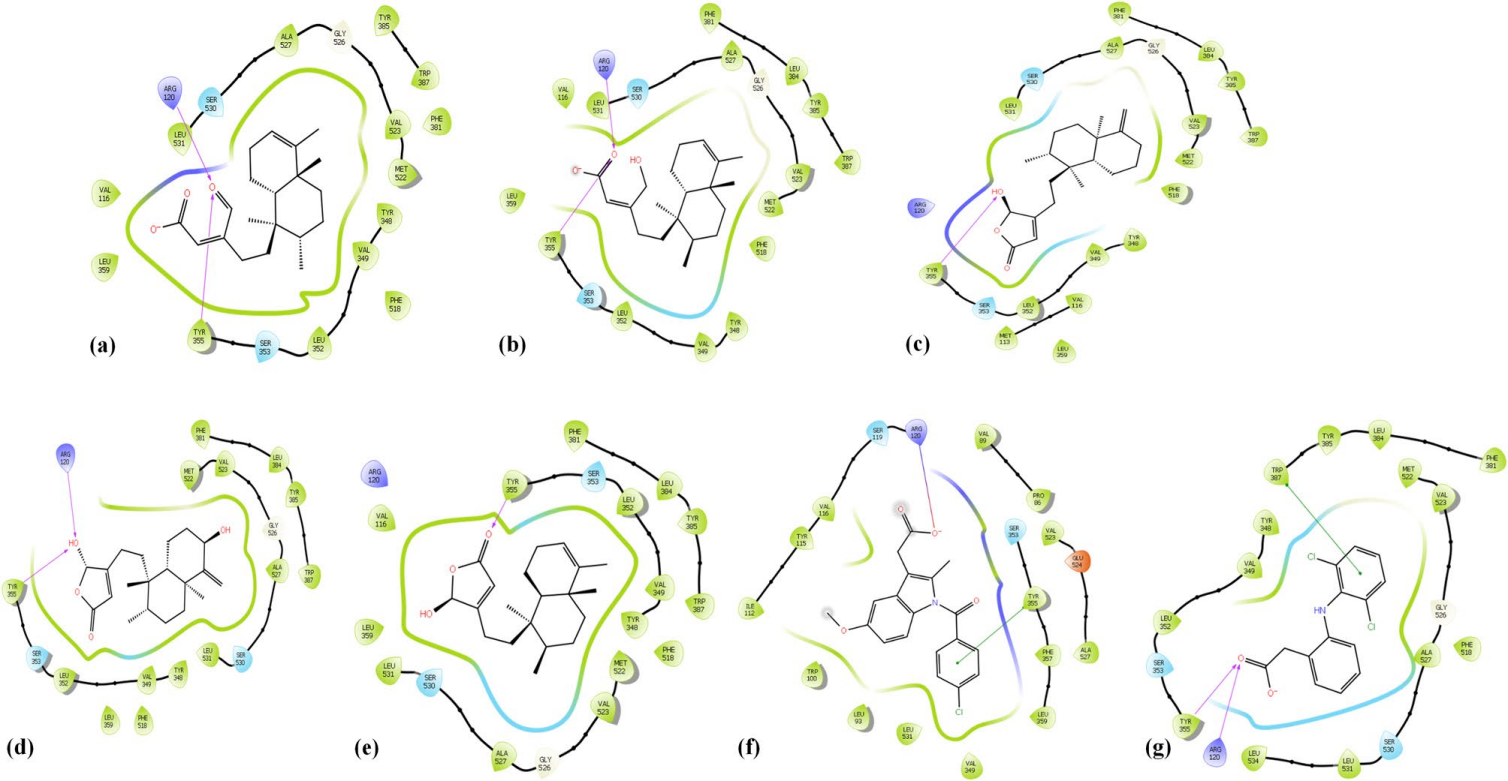
(**a**) Docking pose of compound **1**, (**b**) docking pose of compound **2**, (**c**) docking pose of compound **3**, (**d**) docking pose of compound **4**, (**e**) docking pose of compound **5**, (**f**) docking pose of indomethacin, (**g**) docking pose of diclofenac ligands—with COX-2 (PDB ID: 4COX) protein.

The X-ray crystal structure of the binding sites of COX-1 (PDB ID: 2OYU) and COX-2 (PDB ID: 4COX) specified that the COX-2 binding site was 20% larger than that of COX-1. The approach of ligands to the COX-2 protein was regulated mainly by Val523, Val434, Arg513. Moreover, it has also been well-documented that Arg513 of COX-2 protein acting a vital part with respect to the hydrogen-bond linkage in the COX-2 binding site. In our COX-2 docking studies, we achieved an excellent RMSD of 0.12 Å when native ligand redocking into COX-2 protein. Of which, the compounds **4, 5, 3**, and** 1** showed good COX-2 inhibitor tendance with ΔG_MM-GBSA_ values of − 39.2, − 38.9, − 36.8, and − 30.6 kcal/mol, respectively, which were better as compared to that of the standard drug indomethacin (-29.1 kcal/mol) (Table 1). Compound **2** showed a lower inhibitor capacity with ΔG_MM-GBSA_ of − 15.1 kcal/mol (Table 1). All compounds were well-fixed with the active region of COX-2 protein. Compounds **1, 2,** and **4** formed H-bonds with residues Arg120 and Tyr355, while both compounds **3** and **5** formed one H-bond with Tyr355 (Fig. 5).

**Figure 6:**
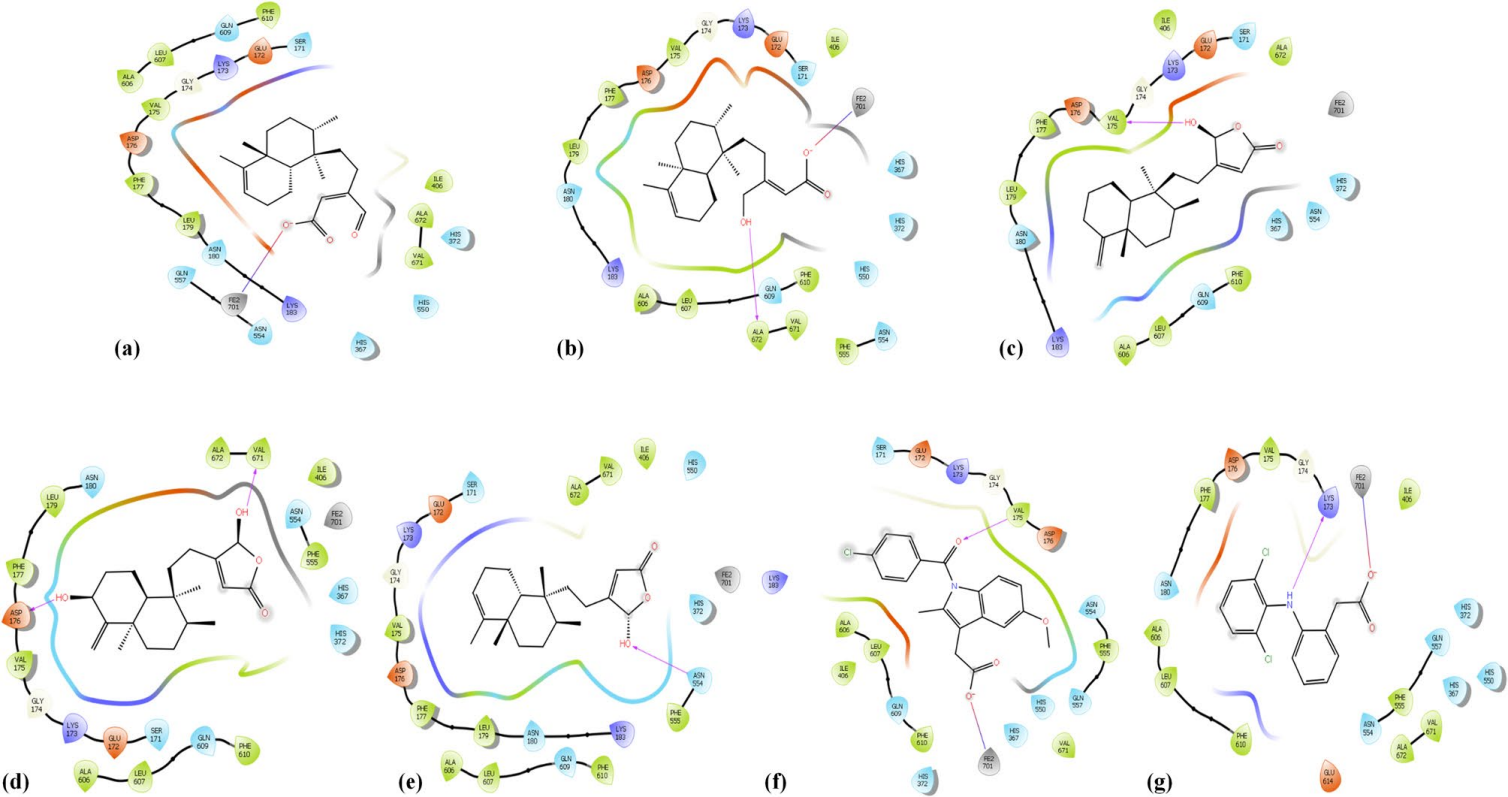
(**a**) Docking pose of compound **1**, (**b**) docking pose of compound **2**, (**c**) docking pose of compound **3**, (**d**) docking pose of compound **4**, (**e**) docking pose of compound **5**, (**f**) Docking pose of indomethacin, (**g**) docking pose of diclofenac ligands—with 5-LOX (PDB ID: 3V99) protein.

Next, we achieved a small RMSD at 0.55 Å of the redocking native ligand into 5-LOX protein, which showed a good-practices experiment of Schrödinger-prepared co-crystallized ligand geometry in comparison to the co-crystallized ligand. Also, all the compounds showed significant interaction with amino acid residues and binding mode of human 5-LOX (PDB ID: 3V99) protein, and forms only one H-bond with 5-LOX enzyme, except compounds **1** and **4**. Compounds **5, 3, 4,** and **2** showed the best prediction for 5-LOX inhibitor in the virtual inspection with ΔG_MM-GBSA_ prediction value of − 50.0, − 42.0, − 35.1, and − 34.0 kcal/mol, respectively, which were stronger than the reference drug diclofenac (− 26.6 kcal/mol) (Table 1). Only compound **1** exhibited a lower estimation to the threshold with ΔG_MM-GBSA_ value of − 18.4 kcal/mol (Table 1). These free energy scores were structurally related to their H-bonding, which was confirmed from their docking poses. The –OH group in compounds **2, 3,** and** 5** formed one H-bond with Ala672, Val175, and Asn554, respectively, while two –OH groups in compound **4** formed H-bonds with Asp176 and Val671. Besides, the –NH group in reference drug diclofenac formed one H-bond with Lys173, while compound **1** has no H-bond interactions due to lack of –OH group (Fig. 6).

Moreover, all compounds **(1–5)** were in the range of 2.2–2.6 Å of the coordination sphere centered by Fe^2+^ ion. The longest distance 2.6 Å to ferrous ion was observed with compound **3**, and indomethacin has the shortest distance with 2.2 Å. Interestingly, the octahedral shape of compound **1**—Fe^2+^ complex should have the most favorable energetic structure as compared to the trigonal bipyramidal shape of other compounds (**2**–**5**) since the former fulfills electron configuration in 3d, 4s, 4p subshell of Fe^2+^ ion. However, its free energy prediction shows a contradiction with a weak ΔG_MM-GBSA_ prediction. This is because compound **1** does not contain any H-bonds, and its hydrophobic part has unfavorable interactions with many polar residues such as Ser171, Glu172, Lys173, Asp176 (Fig. 6a).

### Correlations between ΔG_MM-GBSA_ and ΔG_exp_ values

The obtained correlation coefficient implies the high accuracy of the computations for all three model COX-1/2, and 5-LOX, with a gas constant (R) of 0.81, 0.84, and 0.87, respectively (Figure S19–S21). The experimental binding free energy was determined by using ΔG_exp_ = RT ln*K*_*i*_ whereas gas constant R = 1.987 × 10^−3^ kcal K^−1^ mol^−1^ and absolute temperature T = 300 K. The correlation value of 0.98 shows a high linear dependence between ΔG_exp_ of COX-1/2 (Figure S19). The rendering scatter some important remarks in both cases of COX-1/2 proteins, compounds **3**, **4,** and** 5** proved to be (i) as good as the reference drug indomethacin that could be considered as potential candidates for NSAIDs; (ii) better inhibitors as compared to compounds **1** and **2**; and (iii) no significant differences in ΔG_exp_ values among them, meaning that they could be placed in classical non-selective inhibitors.

## Discussion

Previously, the extracts from *P. longifolia* have been repeatedly noted to have potential anti-inflammatory properties in vivo^21,22^. In this study, we expanded the efforts to identify potential anti-inflammatory agents from the methanolic extracts of seeds of *P. longifolia*. As a result we have identified five known clerodane diterpenes, namely 16-oxo-cleroda-3,13(14)E-dien-15-oic acid **(1)**, 16-hydroxy-cleroda-3,13-dien-15-oic acid **(2)**, 16-hydroxy-cleroda-4(18),13-dien-16,15-olide **(3)**, 3α,16α-dihydroxy-cleroda-4(18), 13(14)Z-dien-15,16- olide **(4)**, and 16α-hydroxy-cleroda-3,13(14)Z-dien-15,16-olide **(5)** (Fig. 1). Initially, all the isolated clerodane diterpenes **(1–5)** were subjected to in vitro studies against COX-1, COX-2, and 5-LOX. According to the results, clerodane diterpenes **3, 4,** and **5** showed better inhibition for both COX/LOX enzymes as compared to the standard drugs, while compound **2** was specifically active against the COX-2 enzyme (Fig. 2). As to our knowledge, this was the first study of clerodane diterpenes on enzymatic inhibitory activities on COX/LOX.

Next, we examined these compounds for their molecular docking studies against COX-1 (PDB ID: 2OYU), COX-2 (PDB: 4COX), and 5-LOX (PDB ID: 3V99) proteins, using Schrödinger software 2020-2 to identify their specificity of inhibition. The computational studies revealed the ability of interaction of clerodane diterpenes **(1–5)** with the pro-inflammatory proteins COX-1/2, and 5-LOX, especially with the primary residues on their catalytic center, which was similar to that of the reference drugs (diclofenac and indomethacin). The maintenance of critical contacts between compounds **3, 4,** and **5** and the tested proteins indicating their fittingness and their potential dual COX/LOX inhibitors (Figs. 3, 4, 5, 6). Particularly, compounds **4** and **5** appeared to be useful COX-1 inhibitors with the ΔG_MM-GBSA_ prediction values better than that of the reference drug indomethacin, while compounds **1, 2,** and **3** showed lower inhibitory activity (Table 1). Besides the number of H-Bonds, the changes in the ligand configuration that help to reduce hydrophobic interactions also play a role. In fact, the open acidic chain in compounds **1**, **2** increased inappropriate interactions with the hydrophobic groups of the biding site as compared to closed hydroxyfuranone chain scaffolds of ligands **3**, **4,** and **5**. This phenomenon was also observed in COX-2 docking results.

The COX-2 docking results revealed that compounds **4, 5, 3**, and** 1** possessed a good COX-2 inhibitory activity, while compound **2** showed the opposite (Table 1). This observation could be partly explained by the fact that the large negative electron density carboxylate group in compound **2** is surrounded by many unfavorable hydrophobic residues. Notably, compounds **1, 2,** and **4** formed two H-bonds with residues Arg120 and Tyr355, while compounds **3** and **5** formed one H-bond with Tyr355 (Fig. 5). These results were consistent with the previous studies on non-steroid anti-inflammatory drugs, which have demonstrated that the interaction with Arg120 must be required for most acidic time-dependent NSAIDs^27,28^.

As compared to the standard drug diclofenac, compounds **5, 3, 4,** and **2** appeared to be better 5-LOX inhibitors, while compound **1** exhibited to have lower inhibition activity. In this case, the formation of hydrogen bonds plays a more critical role than the geometry of ligands in complexes with the ferrous ion. Clearly, the octahedral shape of metal–ligand **1** did not show superiority over the other triangle bipyramidal shapes. However, the fact that all these ligands can create coordination bonds with metal ions is a convincing affirmation for their abilities to inhibit organometallic reactions that can occur between the metal ion and arachidonic acid.

Our in vitro enzymatic inhibitory assay outcomes were compatible with the molecular docking studies in the active sites of the COX-1, COX-2, and 5-LOX enzymes, which both revealed that compounds **3**, **4** and **5** were actively acting against COX/LOX enzymes. Molecularly, these compounds dually inhibit COX/LOX enzymes. Also, compounds **3, 4,** and **5** revealed drug-like effects within those considered suitable for drug candidates. Specifically, the skeleton of clerodane diterpenes could be taken into consideration as a new structural lead that is capacitated for further structural modification and research for the development of a novel class of dual inhibitor of COX/5-LOX enzymes.

Inflammatory reactions were triggered by a higher level of leukotrienes and prostaglandins in the human body, which were produced by COX-1/2, and 5-LOX enzymes, respectively^29^. Prostaglandins were accountable for sensitizing nociceptive fibers, thereby facilitating the passage of a pain stimulus, while through specific integrin, leukotrienes stimulate cell adhesion and neutrophil chemotaxis to vascular endothelium^29^. Therefore, both COX-1/2 inhibitions by anti-inflammatory drugs reduce prostaglandin production, eventually result in the lowering of inflammation and pain. However, particularly inhibition of prostaglandins may lead to the stimulation of the 5-LOX pathway, which end-up with the raise of gastrotoxic leukotrienes. Using dual inhibitors that act against COX-1/2 and 5-LOX help to overcome the side-effects on the gastrointestinal and cardiovascular system caused by COX-1 and selective COX-2 inhibitors, respectively^16^. The novel dual inhibitors, therefore, were holding a particular interest and in urgent need of the management of inflammation. The results from the current study clearly showed that compounds **3, 4,** and **5** could be served as dual COX/5-LOX inhibitors (Fig. 2).

To conclude, the present study was the first report of chemical examination of methanolic extract from the seeds of *P. longifolia*, the significant secondary metabolites present in **ME** were identified as five known clerodane diterpenes **(1–5)**. The in vitro results revealed the potent dual COX/LOX inhibitory activity of compounds **3, 4,** and **5**. In high concordance with the in vitro results, the docking experiments confirmed the high selectivity binding of compounds **3, 4,** and **5** towards all pro-inflammatory enzymes COX-1/2, and 5-LOX and compound **2** selectively towards COX-2. Typically, in the COX-1 protein, the gate-holder residues: Ser516, Ile517, and Phe518 often involved in creating H-bonds with most carboxylate and hydroxy groups of the compounds. In COX-2, on the other hand, Arg120 and Tyr355 were critical residues for binding mode of COX-2 and essential for H-bond formation. In the case of 5-LOX, the coordination bonds the metal ion were first established, followed by the H-bonds that helped to regulated the binding of ligands. Taken together, this study uncovered three promising anti-inflammatory agents with high potency against COX and LOX that can be served in anti-inflammatory drug discovery and development.

## Methods

### Plant material

The seeds of *Polyalthia longifolia* (Sonn.) Thwaites were collected from Seshachalam hills, Tirupati, India, in 2019. The sample has been authenticated and a voucher specimen (PS-2019-349) has been deposited at the Department of Botany, Sri Venkateswara University, India.

### Extraction and isolation

The powder of dried seeds of *P. longifolia* (1.0 kg) was extracted with absolute methanol at room temperature (3 times). The whole extract was evaporated under low pressure to obtain a methanolic extract (**ME**, 45 g, 4.5% w/w)^30^. By using column chromatography (CC) of mesh size 100–200 via a hexane/ethyl acetate solvent system (step gradient flow), **ME** extract (5.0 g) yielded five major fractions (F1-5). F1 fraction (2 g) was subjected to CC using the above parameters yielded **1** (110 mg) as a colorless gum. By using step gradient flow dichloromethane/ethyl acetate solvent system, F2 (2.5 g) gave **2** (115 mg) as a brownish gum, F3 (800 mg) yielded **3** (80 mg) as a colorless oil, F4 (1.0 g) yielded **4** (100 mg) as a white solid, and F5 (1.2 g) yielded **5** (120 mg) as a colorless gum.

### In vitro assays of anti-inflammatory activity

#### Cyclooxygenase (COX-1 and COX-2) inhibitory assay

The abilities of five metabolites **(1–5)** to inhibit isoenzymes COX-1/2 were performed using COX (ovine/human) inhibitor assay kit (Cayman, No.: 560131)^31^. To 10 µl of either COX1 or COX2 added 0.1 M Tris–HCl buffer (960 µl) and different known concentrations of test samples and incubated at 37 °C for 10 min. Later 10 µl of 100 µM arachidonic acid, after 2 min 1 M HCl of 50 µl and Ellman’s reagent, were added. The absorbance was measured spectrophotometrically at 410 nm against the blank. The percentage of inhibition was deliberated with the absorbance values, and IC_50_ values were calculated by linear regression.

#### 5-lipoxygenase inhibitory assay

The five metabolites **(1–5)** were tested against 5-LOX (human recombinant) using 5-LOX assay kit (No. 437996, Sigma Aldrich)^17^. To 90 µl of 5-LOX enzyme solution added different test sample concentrations, 100 µl of de chromogen, and finally added 10 µl of the substrate (arachidonic acid) and gently shake or 10 min and absorbance was measured at 490 nm against the blank. The % inhibition was deliberated with the absorbance values by which IC_50_ values were calculated by linear regression.

### In silico study

#### Computer and software

The in silico docking, MM-GBSA free binding energy evaluations, and rendering model outputs were performed using Schrödinger software 2020-2. The scatter plots of correlation figures were designed by Tableau 2020.2.

#### Docking and scoring

Docking calculations were performed with the Glide program^32^. In fact, all the prepared ligands in previous steps were docked and scored firstly with Standard-precision (SP). The top-scored SP results of each ligand were redocked with higher-level Extra-precision (XP) that help to eliminate the false positive case and to engage a good enrichment. Nevertheless, the general purpose of Glide-scoring functions is to achieve high throughput screening a large number of compounds feasible by simplifying the calculation with different approximations. So, this procedure shows only the reliable and correct binding mode with the lower RMSD of native co-crystallized structures, deducing that the higher SP or XP values do not always go along with the better free binding energies. Therefore, a complete free binding energy estimation, usually the dynamic integration (TI), free energy perturbation (FEP+), or molecular mechanics with generalized Born and surface area (MM-GBSA) methods^33^, need to be conducted after Glide scoring^34^. However, MM-GBSA is usually selected due to the fact that it uses the fastest force-field based method to calculates the free binding energy.

#### Binding affinity calculations

The follow-up of Glide docking is a binding free energy prediction via the Molecular Mechanics/Generalized Born and Surface Area method (MM-GBSA)^33,35^. This free energy is defined by the difference of the complex with the specific energy of protein and ligand (Eq. 1). This total energy has its heat of reaction estimated by the addition of the Entropy terms (ΔS), the solvation free energy (ΔG_sol_), and the gas-phase interaction energy (ΔE_gas_) (Eq. 2). Usually, at constant pressure, the heat of reaction is almost equivalent to the change of internal energy, so the (ΔE_int_) is canceled (Eq. 3). The rest forms of energy include all intermolecular interactions such as electrostatic interactions, protein–ligand vdW contacts, ligand desolation, and internal strain energies with OPLS2005 force field. Next to the heat of reaction, the solvation free energy (ΔG_sol_) is sub-categorized into the non-polar (ΔG_Surf_) and polar (ΔG_GB_) energy forms and their solvation energy is calculated by using the solvent-accessible surface area and GB model, respectively (Eq. 4). Briefly, the binding free energy (ΔG_bind_) is calculated by the addition of the solvation free energy and gas-phase interaction energy. At the same time, the entropy term is neglected in the calculation for relative free binding energies^34^.

1 $$\Delta G_{bind} = G_{complex} - G_{receptor} - G_{ligand}$$

2 $$\Delta G_{bind} = \Delta H - T\Delta S \approx \Delta E_{gas} + \Delta G_{sol} - T\Delta S$$

3 $$\Delta E_{gas} = \Delta E_{int} + \Delta E_{ELE} + \Delta E_{VDW}$$

4 $$\Delta G_{sol} = \Delta G_{GB} + \Delta G_{Surf}$$

Consequently, the MM-GBSA calculations are not in agreement with experimental binding affinities, but they show the tendency to bind and the reasonable correlation with the experiment values, and the more negative value MM-GBSA indicates more potent approximate free energies of binding. Otherwise, MM-GBSA is implemented directly in the Prime module (version 3.1, Schro¨dinger, LLC, New York, NY, 2012) from Schrodinger Suite using default system and protein–ligand complexes were extracted from top scores XP dockings.

### Statistical analysis

All in vitro assay results were noted as mean ± SD. One-way analysis of variance (ANOVA) followed by Student’s t-test and *p* < 0.05 was considered to be statistically significant.

## Supplementary information

Supplementary file1.
