# Supplementary material for: Dual COX and 5-LOX inhibition by clerodane diterpenes from seeds of Polyalthia longifolia (Sonn.) Thwaites
Source: Sci Rep. 2020 Sep 29;10:15965. doi: 10.1038/s41598-020-72840-8 (PMC7524750; doi:10.1038/s41598-020-72840-8)
Supplement: Supplementary file 1 — Supplementary file1. [file 41598_2020_72840_MOESM1_ESM.doc]

**Supporting Information to**

# Dual COX and 5-LOX inhibition by clerodane diterpenes from seeds of *Polyalthia longifolia* (Sonn.) Thwaites

# Ha Thi Nguyen^1,2^, Thien-Y Vu^3^, Vishala Chandi^4^, Haritha Polimati^4,5^, and Vinay Bharadwaj Tatipamula^1,2,*^

^1^Institute of Research and Development, Duy Tan University, Da Nang 550000, Vietnam

^2^Faculty of Medicine, Duy Tan University, Danang 550000, Vietnam

^3^Faculty of Pharmacy, Ton Duc Thang University, Ho Chi Minh City 700000, Vietnam

^4^Pharmacology Department, AU College of Pharmaceutical Sciences, Andhra University, Visakhapatnam 530003, Andhra Pradesh, India

^5^Pharmacology and Toxicology Division, Incozen Therapeutics Pvt Ltd., Turkapally, Telangana 500078, India

*[vinaybharadwajtatipamula@duytan.edu.vn](mailto:vinaybharadwajtatipamula@duytan.edu.vn)

**Materials and Methods**

***In silico study***

*Ligand preparation:* Five molecules **1-5** were created by 2D sketcher and treated by Ligprep (Schrödinger, LLC, New York, NY, 2020) procedures, whereas the possible states at physiological pH = 7.0
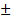
 2.0 were proposed by Epik^1,2^ and all 3D combinations were generated up to 32 stereoisomers per ligand.

*Protein preparation:* The crystal structure of 5-LOX protein downloaded from Protein Data Bank (PDB ID: [3V99](https://www.rcsb.org/structure/3V99)) was imported and prepared by Protein Preparation Wizard^3^ workflow. Firstly, the raw structure was preprocessed by default options to add missing hydrogens, to delete waters beyond 5 Å from the het groups, or to generate suitable states for protein residues at physiological pH 7.0 ± 2.0. The pre-processed protein was then optimized and removed all the waters with less than 4 H-bonds. Finally, the protein was minimized to free all hydrogen atoms while the heavy atoms were converged to root-mean-square deviation (RMSD) of 0.30 Å. Similarly, COX-1 (PDB ID: [2OYU](https://www.rcsb.org/structure/2OYU)) and COX-2 protein (PDB ID: [4COX](https://www.rcsb.org/structure/4COX)) were prepared using the same procedure. None of the water molecules of the three proteins were observed to form a bridge of H-bonds between ligands and proteins, indicating that all water molecules were successfully removed.

*Receptor grid generation:* The grid box for the docking job of all three proteins was centered by the ligand in the crystal structure, and its dimension was given by 20 Å × 20 Å × 20 Å. The constraints differ depending on the nature of each protein.

*COX-1 grid box and COX-2 grid box:* COX-1 and COX-2 are isoenzymes. Their similarity in overall structure and active sites are responsible for their resemblance in catalytic mechanisms, kinetics, and end products. However, COX-2 has a larger binding site mostly due to the differences in three amino acids at positions 434, 513, 523 (Ile, His, Ile for COX-1 vs. Val, Arg, Val for COX-2, respectively) that could be exploited in developing COX-2-specific inhibitors (Figure S16)^4^. The COX-1 and COX-2 grid box must mainly cover twenty-four residues of hydrophobic cyclooxygenase in their active site. In which, Ser530 is the acetylation target by aspirin^5,6^, and Arg120 is a binding site for carboxylate groups of fatty acids and many NSAIDs^7^.

*5-LOX grid box:* 5-LOX protein is generally recognized as the catalyst for both oxidation reaction of arachidonic acid to 5-HpETE and the dehydration reaction of 5-HpETE to leukotriene-A_4_ (Figure S17)^8^. Chemically, the ferric ion (Fe^3+^) cofactor with hydroxide ion OH^–^ started the organometallic reaction by an abstraction of a hydrogen atom at C-7 of AA and an insertion of O_2_ at C-5 to generate (5S)-HpETE. The second redox cycle will remove 10 pro-R hydrogen atoms from (5S)-HpETE, and then the double bonds of the reactant will rearrange to form leukotriene-A_4_. The final state of iron ion is +2 corresponding to the changement of ligand type X (OH^–^) to ligand type L (H_2_O)^9,10^. Structurally, the ferrous ion is held principally into place by three coordination with His residues (numbered 367, 372 and 550), it may also be supported by Asn554 residue via a water molecule 909 (crystal structure 3V99 of 5-LOX), but Asn554 is not close enough for a coordination sphere. Consequently, the volume of the grid box includes mainly ferrous ion, and the “metal constraint” will be established for Fe^2+^. This constraint must be matched for all ligands and reference drugs to guarantee that they act as vertices in the complex Fe^2+^ ion.

**Results**

**Proton NMR, ^13^C NMR and ESI-MS results for five isolated compounds**

**
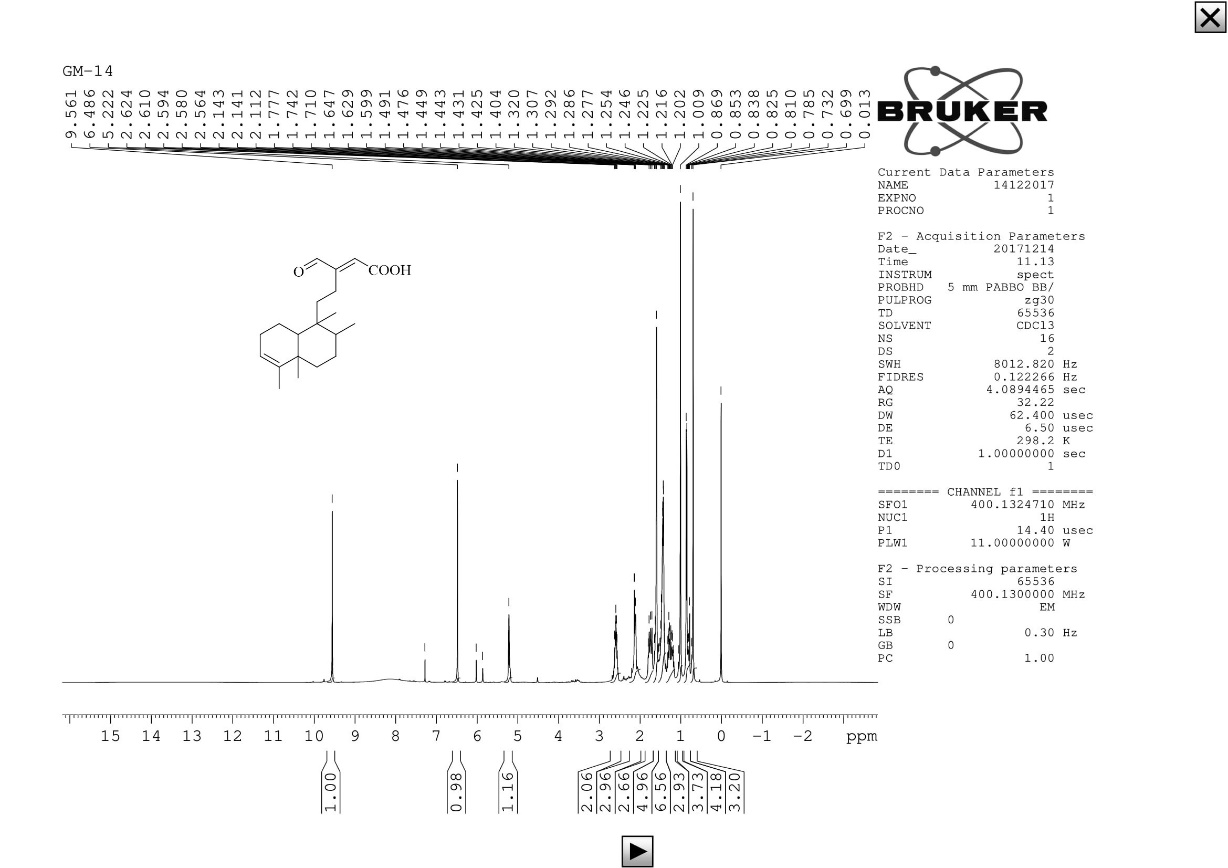
**

**Figure S1.** Proton NMR of **1** (400 MHz, CDCl_3_)


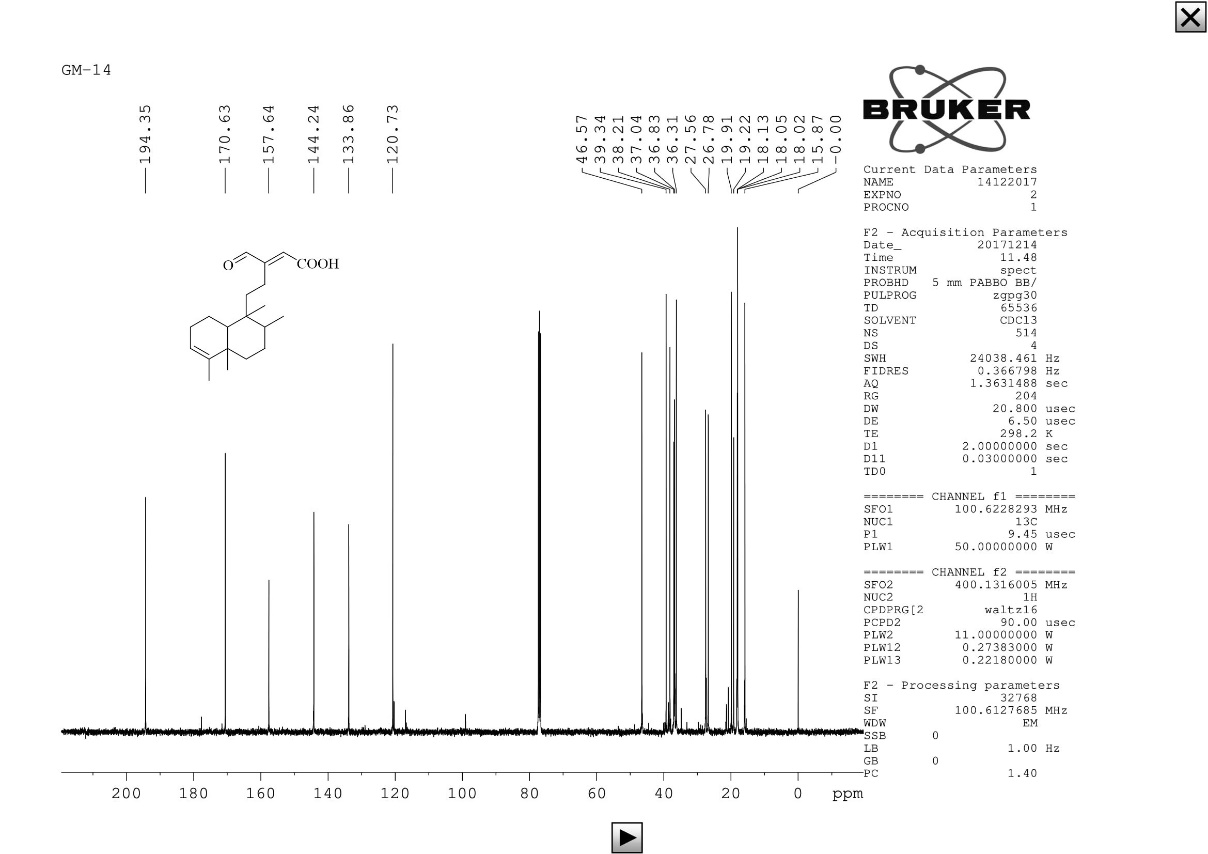


**Figure S2.** ^13^C NMR of **1** (400 MHz, CDCl_3_)


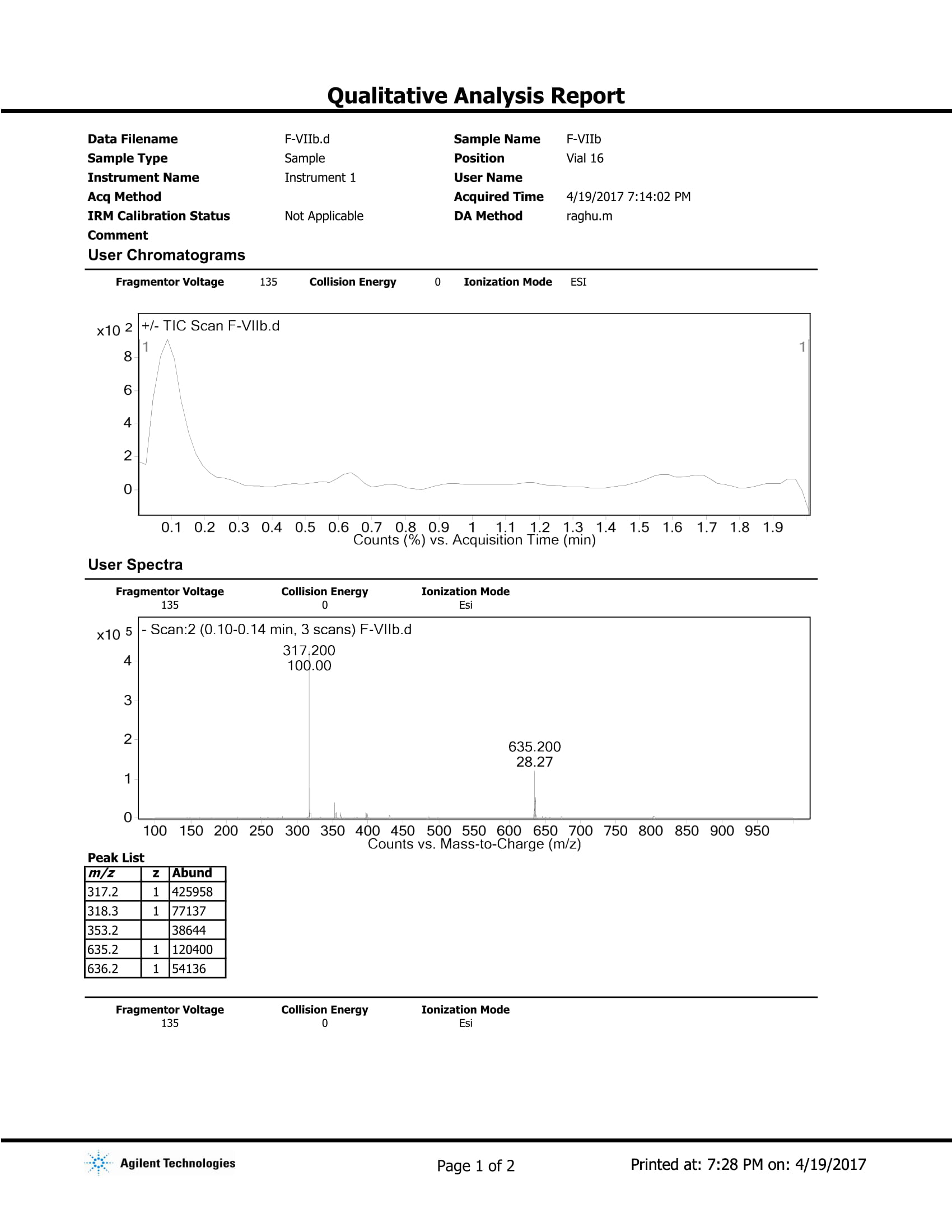


**Figure S3.** ESI-MS of **1** (negative mode)


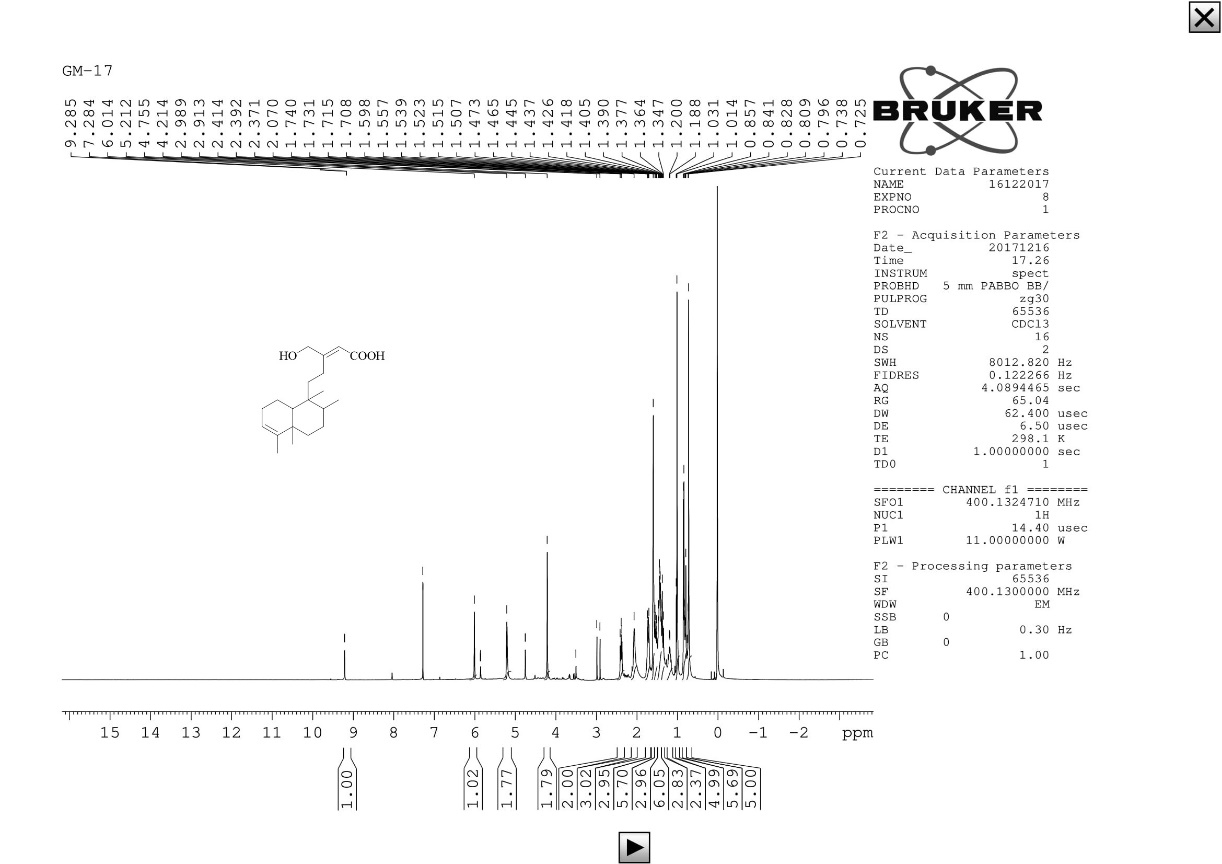


**Figure S4.** Proton NMR of **2** (400 MHz, CDCl_3_)


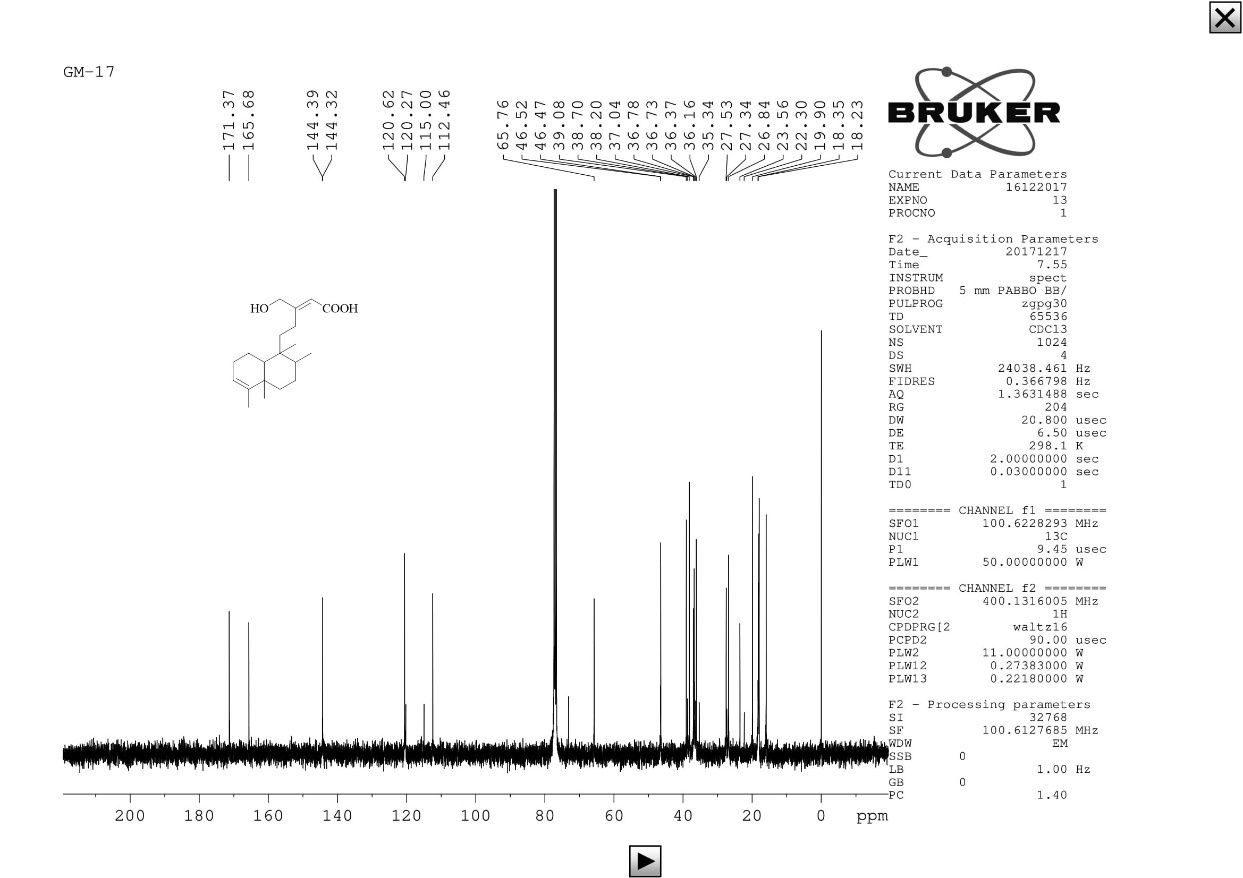


**Figure S5.** ^13^C NMR of **2** (400 MHz, CDCl_3_)


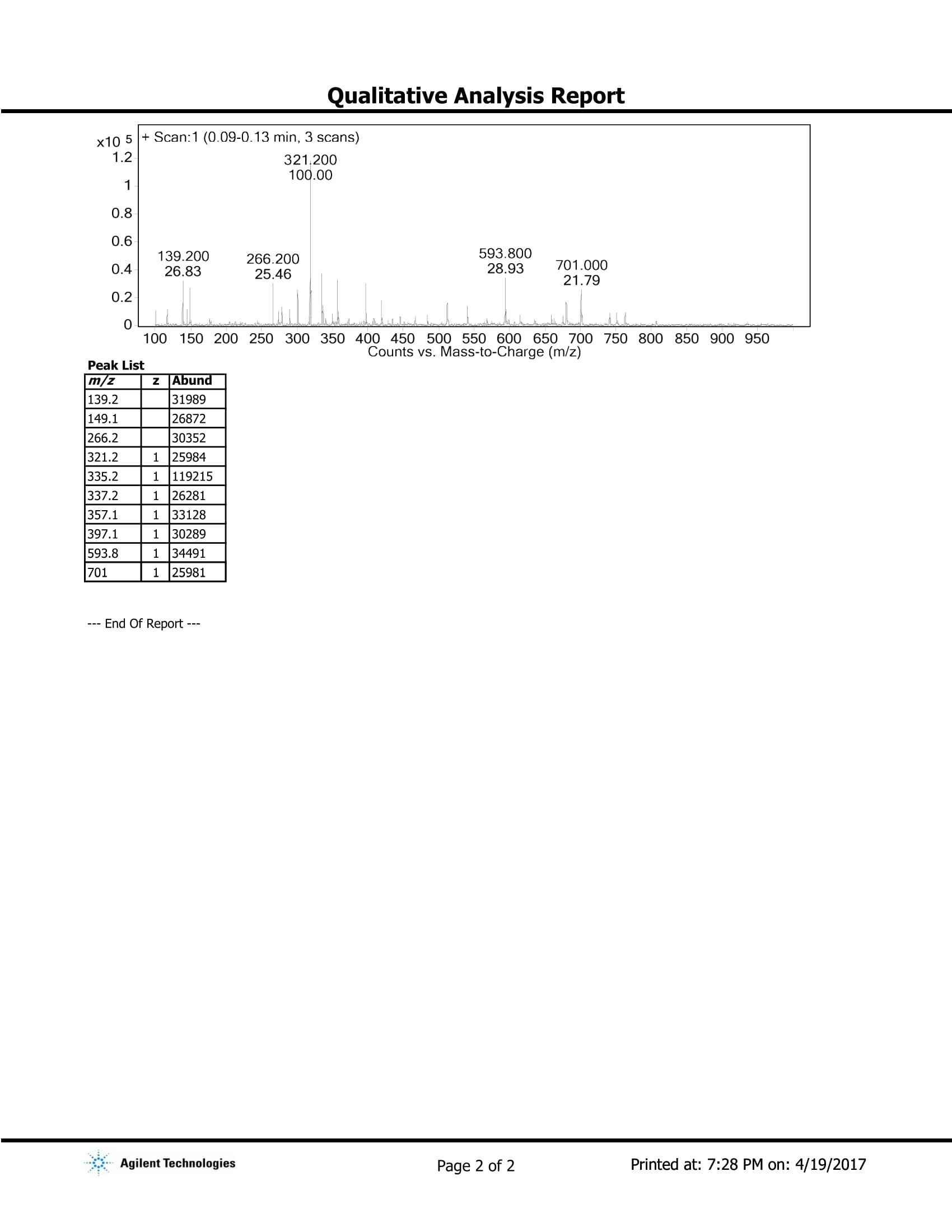


**Figure S6.** ESI-MS of **2** (positive mode)


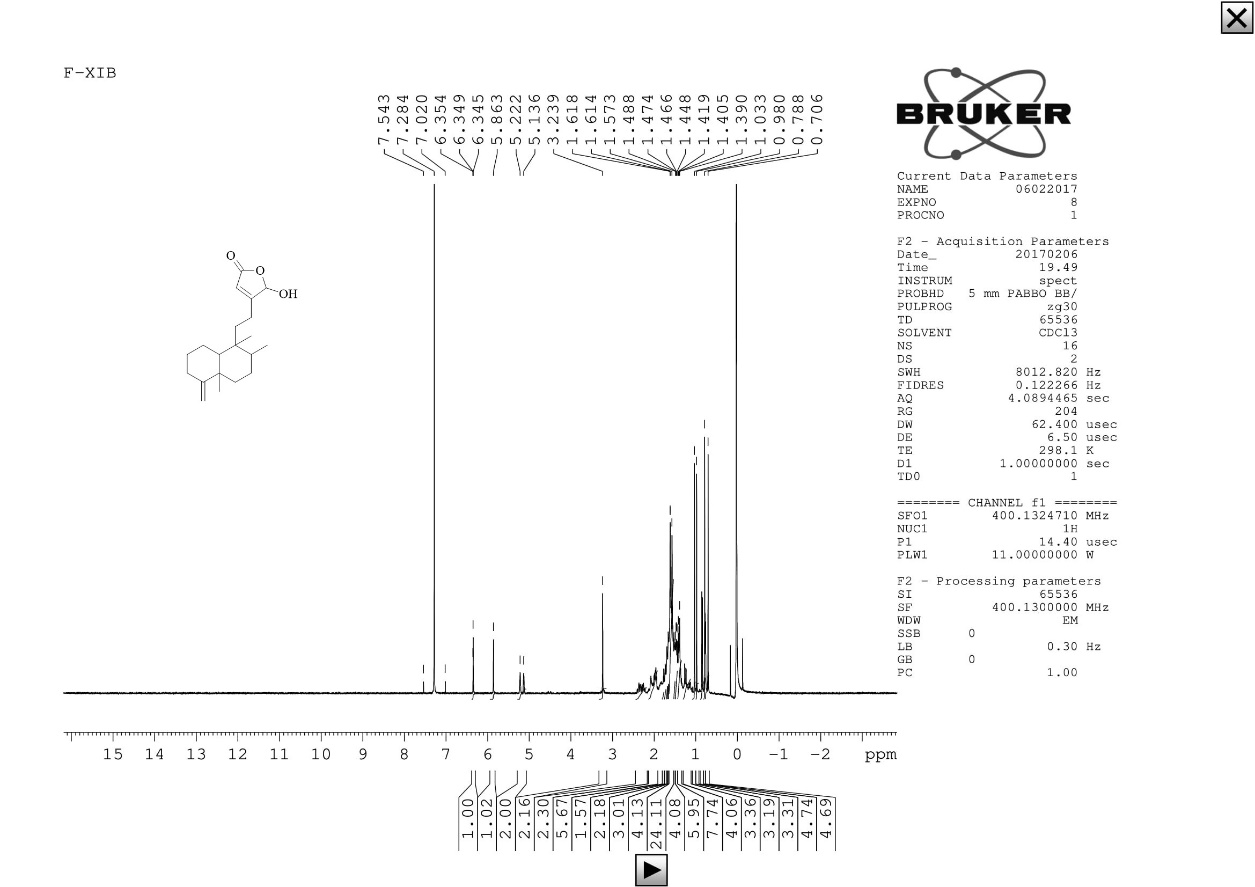


**Figure S7.** Proton NMR of **3** (400 MHz, CDCl_3_)


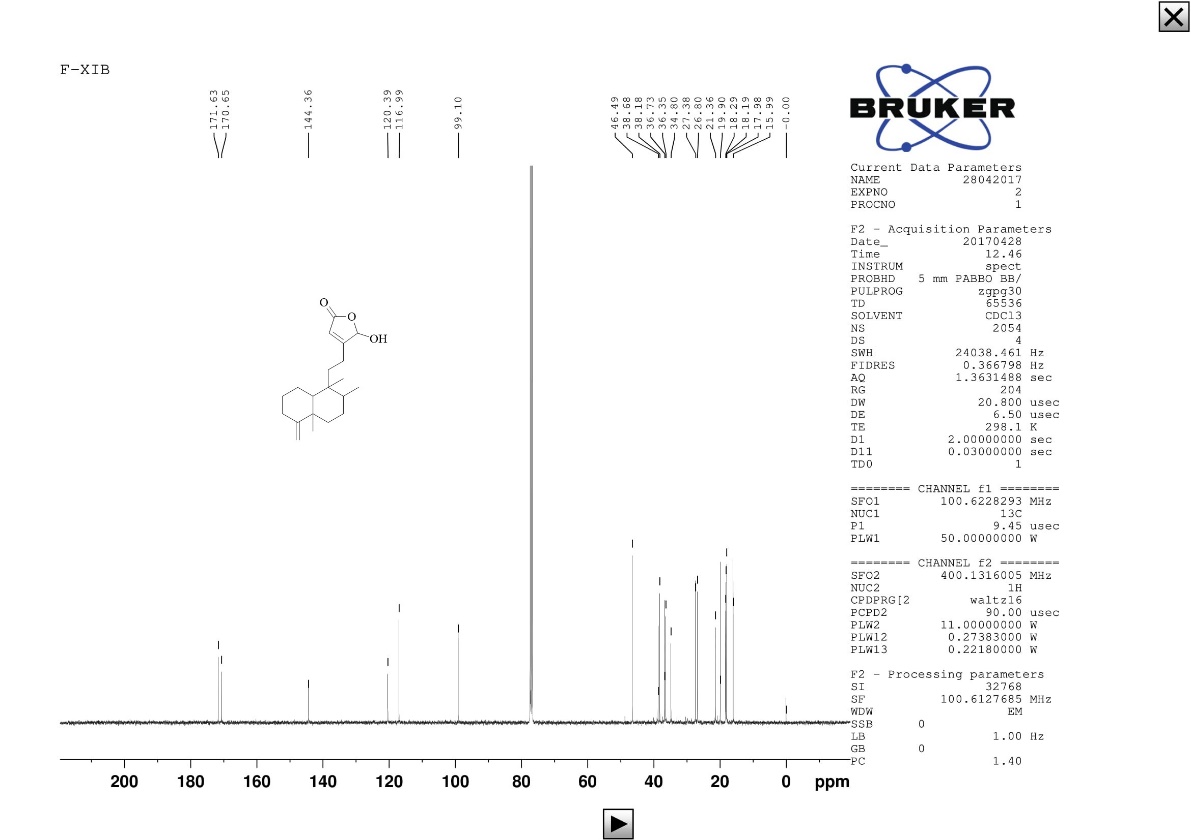


**Figure S8.** ^13^C NMR of **3** (400 MHz, CDCl_3_)


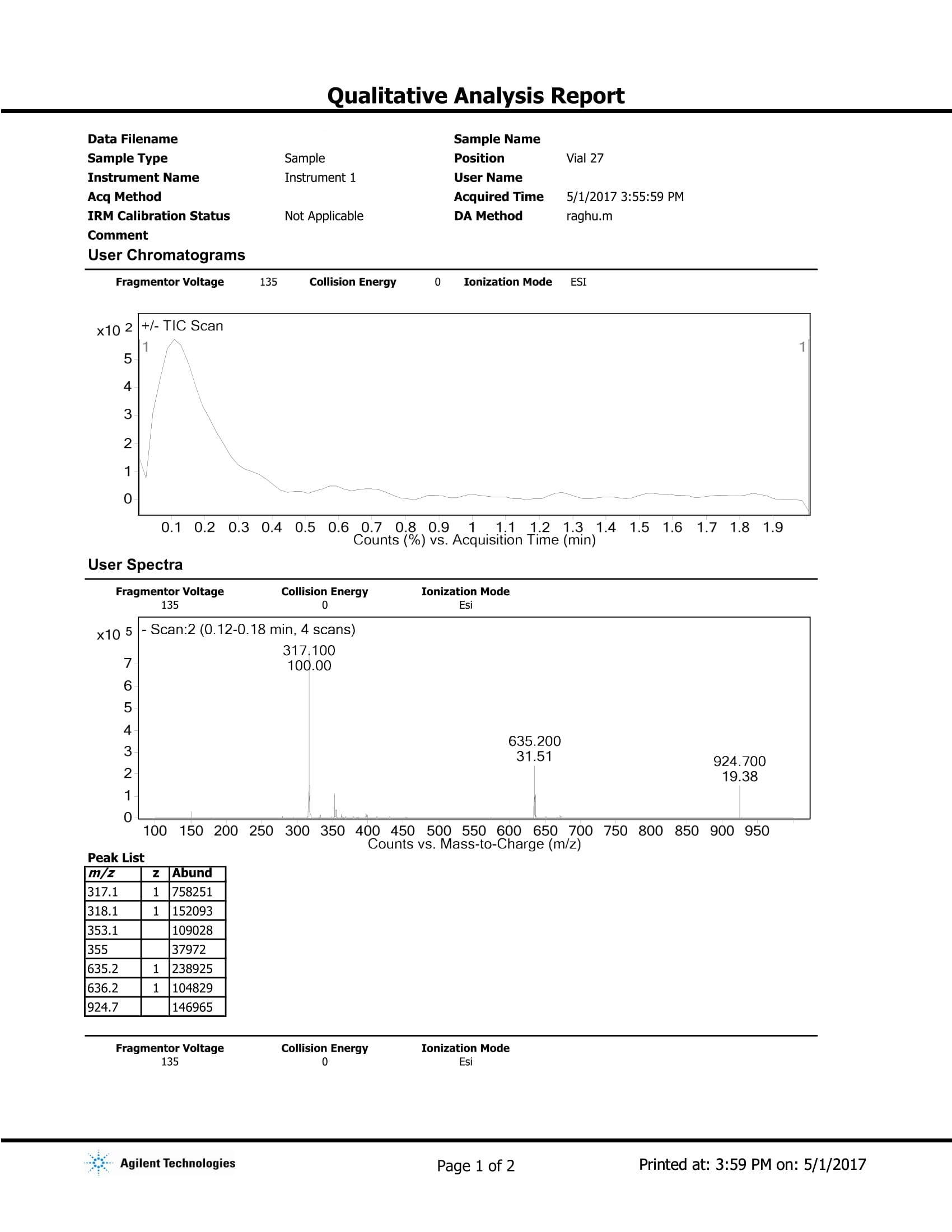


**Figure S9.** ESI-MS of **3** (negative mode)


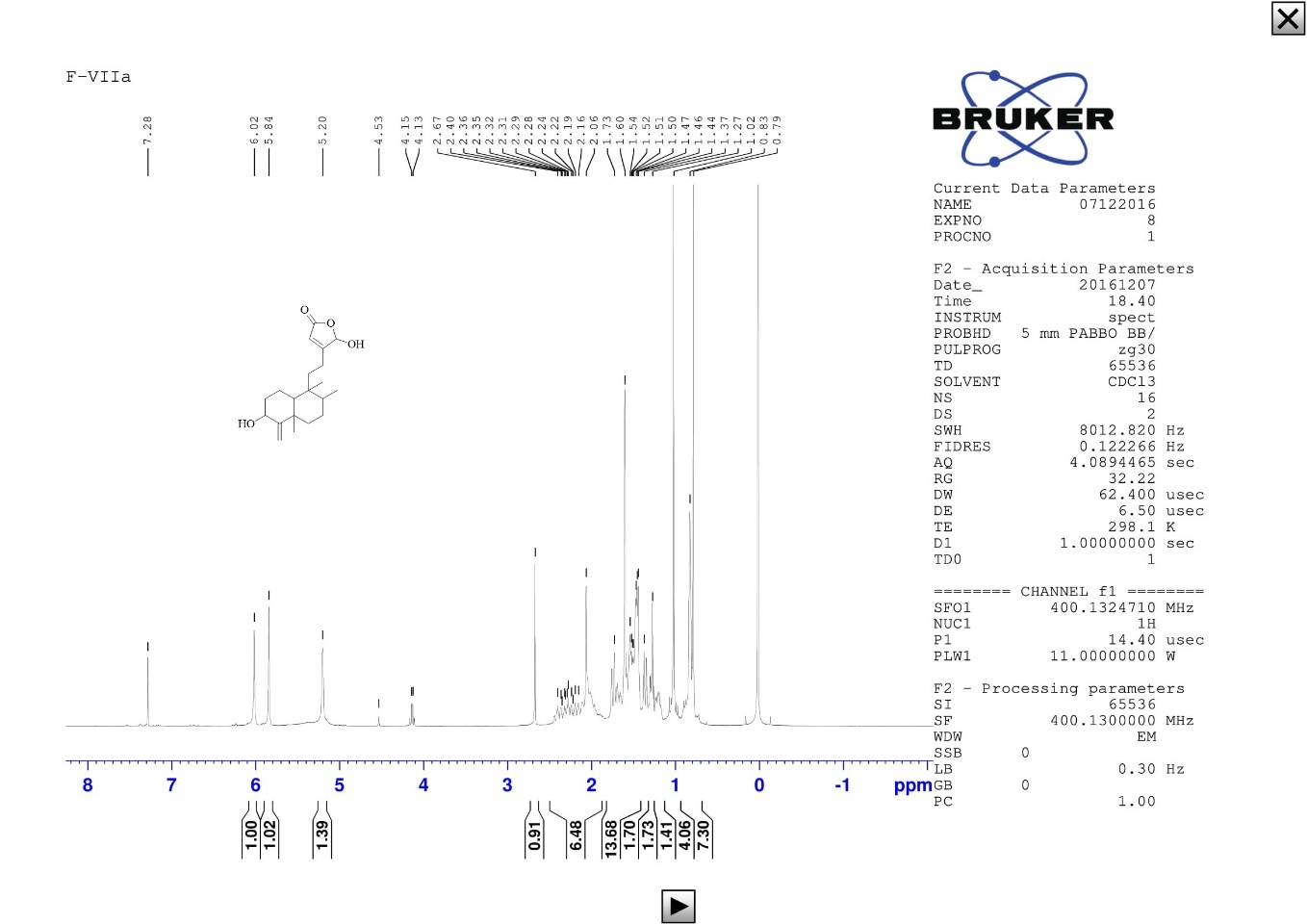


**Figure S10.** Proton NMR of **4** (400 MHz, CDCl_3_)


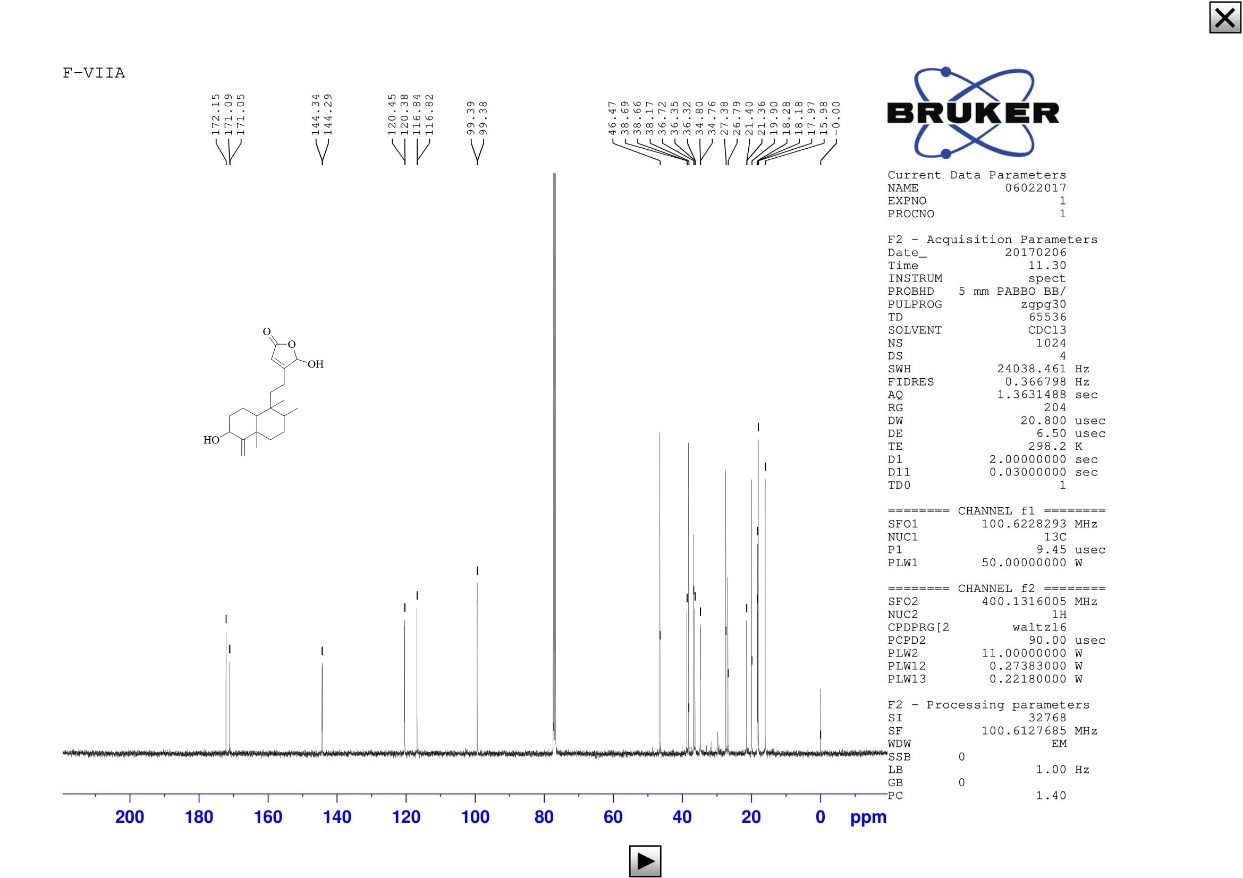


**Figure S11.** ^13^C NMR of **4** (400 MHz, CDCl_3_)


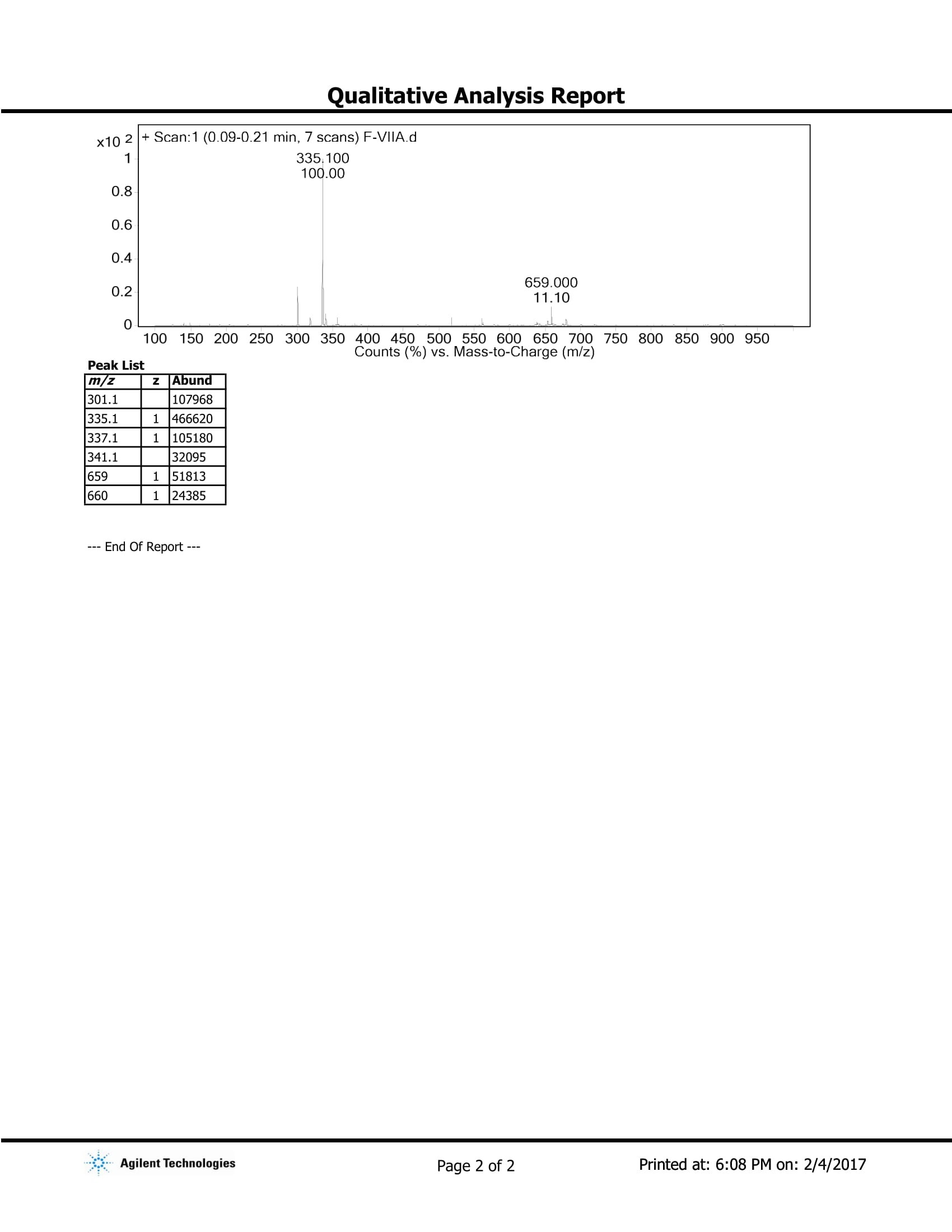


**Figure S12.** ESI-MS of **4** (positive mode)


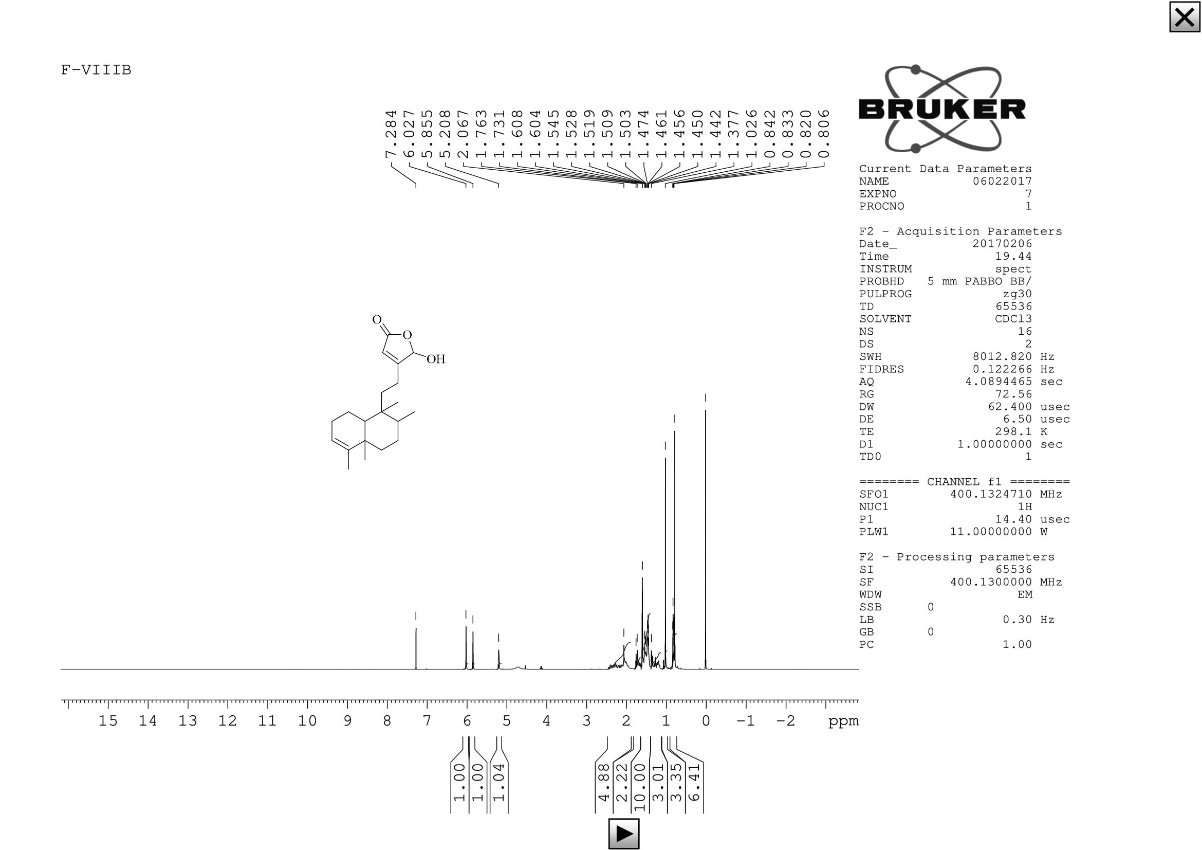


**Figure S13.** Proton NMR of **5** (400 MHz, CDCl_3_)


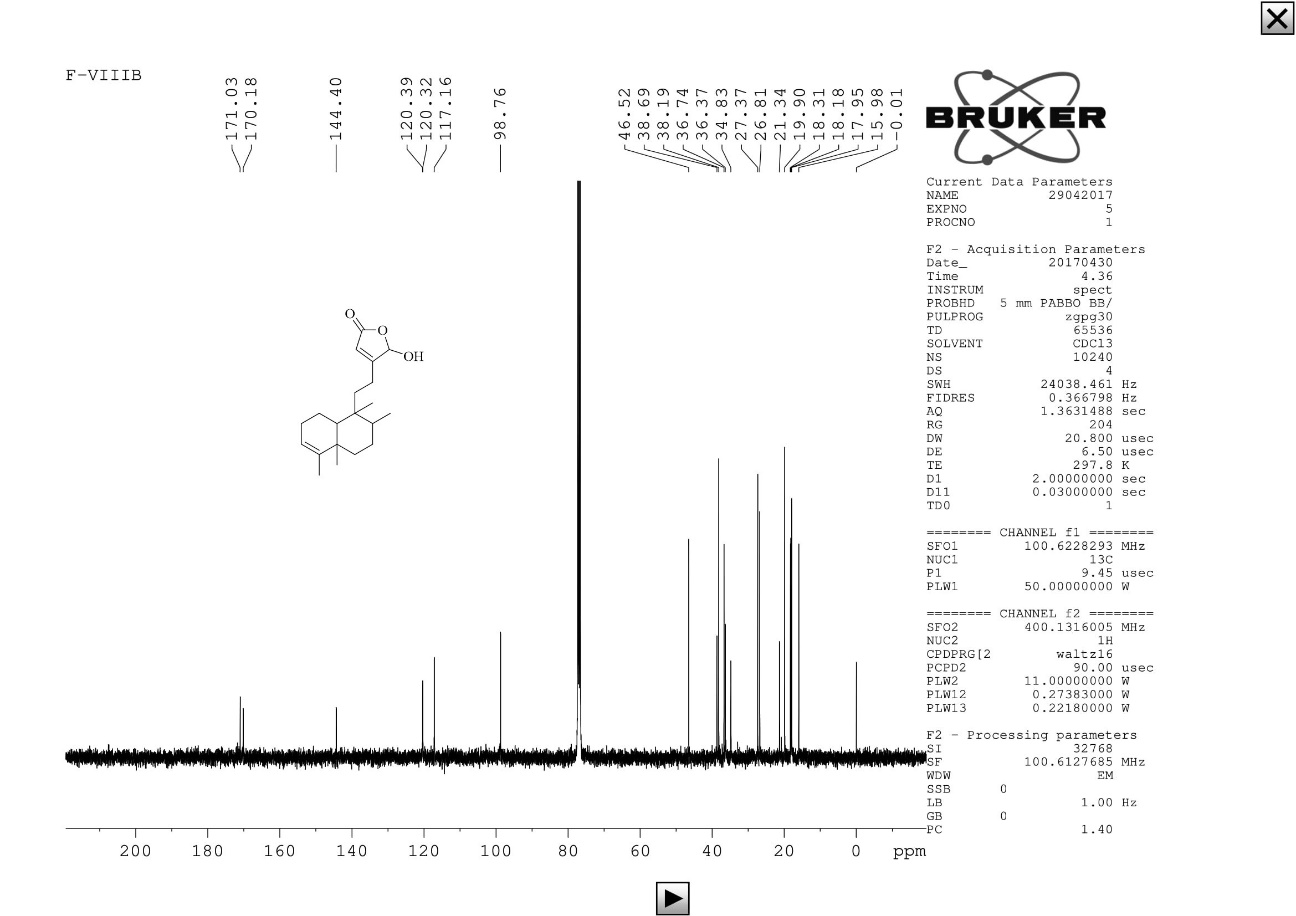


**Figure S14.** ^13^C NMR of **5** (400 MHz, CDCl_3_)


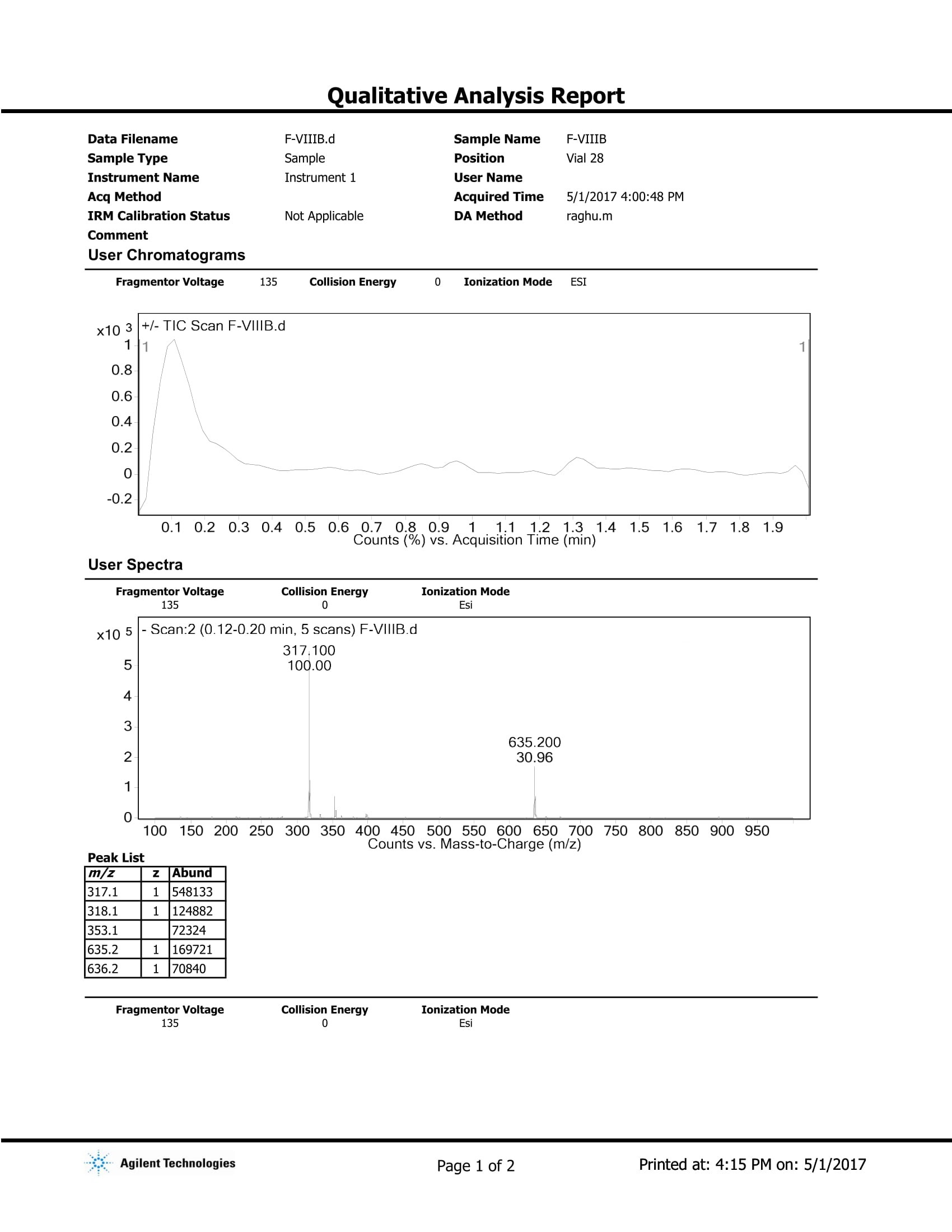


**Figure S15.** ESI-MS of **5** (negative mode)

**Characterization of compounds (1-5) from methanolic extract of seeds of *Polyalthia longifolia***

***Compound 1 (16-oxo-cleroda-3,13(14)E- dien-15-oic acid)***^11^ ***characterisation data:*** mp: 299-300 ˚C; TLC (hexane:ethyl acetate, 1:1 v/v): R_f_ = 0.6; ^1^H NMR (400 MHz, CDCl_3_): δ 9.561 (s, 1H), 6.486 (s, 1H), 5.222 (s, 1H), 2.564-2. 624 (m, 2H), 2.141-2.143 (d, J= 0.8 Hz, 2H), 2.112 (s, 1H), 1.629-1.802 (m, 1H), 1.561-1.599 (m, 1H), 1.550 (s, 3H), 1.404-1.512 (m, 6H), 1.172-1.292 (m, 2H), 1.009 (s, 3H), 0.869-0.853 (d, J= 6.4 Hz, 3H), 0.699 (s, 3H); ^13^C NMR (400 MHz, CDCl_3_): δ 194.35, 170.63, 157.64, 144.24, 133.86, 120.73, 46.57, 39.34, 38.21, 37.04, 36.83, 36.31, 27.56, 26.78, 19.91, 19.22, 18.13, 18.05, 18.02, 15.87; UV (Methanol): λ_max_ 238; ESI-MS *m/z*: [M]^+^ calcd. for C_20_H_30_O_3_: 318.22; found, 317.200 ([M]^+^ - [H]^+^, 100%, negative mode).

***Compound 2 (16-hydroxy-cleroda-3,13-dien-15-oic acid)***^12^ ***characterisation data:*** mp: 318-319 ˚C; TLC (CH_2_Cl_2_:ethyl acetate, 1:1 v/v): R_f_ = 0.4; ^1^H NMR (400 MHz, CDCl_3_): δ 9.285 (s, 1H), 6.014 (s, 1H), 5.212 (s, 1H), 4.214 (s, 2H), 2.913-2.989 (m, 3H), 2.371-2.414 (m, 4H), 1.740 (s, 1H), 1.507-1.598 (m, 2H), 1.200-1.473 (m, 5H), 1.014 (s, 3H), 0.841 (s, 3H), 0.828 (s, 3H), 0.725 (s, 3H); ^13^C NMR (400 MHz, CDCl_3_): δ 171.37, 165.68, 144.39, 120.62, 112.46, 65.76, 46.52, 39.08, 38.70, 37.04, 36.73, 35.34, 27.53, 27.34, 26.84, 23.56, 23.30, 19.90, 18.35, 18.23; UV (Ethanol): λ_max_ 256; ESI-MS *m/z*: [M]^+^ calcd. for C_20_H_32_O_3_: 320.24; found, 321.200 ([M]^+^ + [H]^+^, 100%, positive mode).

***Compound 3 (16-hydroxy-cleroda-4(18),13-dien-16,15-olide)***^13^ ***characterisation data:*** mp: 250-251˚C; TLC (hexane:ethyl acetate, 1:1 v/v): R_f_ = 0.4; ^1^H NMR (400 MHz, CDCl_3_): δ 6.349 (s, 1H), 5.863 (s, 1H), 5.136-5.222 (d, J= 34.4 Hz, 2H), 3.239 (s, 1H), 1.614-1.618 (m, 5H), 1.573 (m, 2H), 1.448-1.488 (m, 5H), 1.390-1.419 (m, 4H), 0.980 (s, 3H), 0.788 (s, 3H), 0.706 (s, 3H); ^13^C NMR (400 MHz, CDCl_3_): δ 171.63, 170.65, 144.36, 120.39, 116.99, 99.10, 46.49, 38.68, 38.18, 36.73, 36.35, 34.80, 27.38, 26.80, 21.36, 19.90, 18.29, 18.19, 17.98, 15.99; UV (Ethanol): λ_max_ 215; ESI-MS *m/z*: [M]^+^ calcd. for C_20_H_30_O_3_: 318.22; found, 317.100 ([M]^+^ - [H]^+^, 100%, negative mode).

***Compound 4 (3α,16α-dihydroxy-cleroda-4(18), 13(14)Z-dien-15,16- olide)***^14^ ***characterisation data:*** mp: 307-308 ˚C; TLC (hexane:CH_2_Cl_2_, 1:1 v/v): R_f_ = 0.6; ^1^H NMR (400 MHz, CDCl_3_): δ 6.02 (s, 1H), 5.84 (s, 1H), 4.13-4.15 (m, 2H), 5.20 (s, 1H), 2.67 (s, 1H), 2.19-2.40 (m, 7H), 2.16 (s, 1H), 1.60-1.73 (m, 2H), 1.50-1.54 (m, 2H), 1.44-1.47 (m, 1H), 1.27-1.37 (m, 2H), 1.02 (s, 3H), 0.83 (s, 3H), 0.79 (s, 3H); ^13^C NMR (400 MHz, CDCl_3_): δ 172.15, 171.05, 144.34, 144.29, 120.38, 116.82, 99.38, 46.47, 38.69, 38.17, 36.72, 36.32, 34.76, 26.79, 21.36, 19.90, 18.28, 18.18, 17.97, 15.98; UV (Ethanol): λ_max_ 210; ESI-MS *m/z*: [M]^+^ calcd. for C_20_H_30_O_4_: 334.21; found, 335.100 ([M]^+^ + [H]^+^, 100%, positive mode); analysis (calcd., found for C_20_H_30_O_4_): C (71.63, 71.82), H (9.08, 9.04).

***Compound 5* *(16α-hydroxy-cleroda-3,13(14)Z-dien-15,16-olide)***^11^ ***characterisation data:*** mp: 249-250 ˚C; TLC (hexane:ethyl acetate, 1:1 v/v): R_f_ = 0.3; ^1^H NMR (400 MHz, CDCl_3_): δ 6.027 (s, 1H), 5.855 (s, 1H), 5.208 (s, 1H), 2.067 (m, 4H), 1.763 (m, 2H), 1.731 (s, 1H), 1.377-1.608 (m, 8H), 1.206 (s, 3H), 0.833 (s, 3H), 0.820 (s, 3H), 0.806 (s, 3H); ^13^C NMR (400 MHz, CDCl_3_): δ 171.03, 170.18, 144.40, 120.39, 117.16, 98.76, 46.52, 38.69, 38.19, 36.74, 36.37, 34.83, 27.37, 26.81, 21.34, 19.90, 18.31, 18.18, 17.95, 15.98; UV (Ethanol): λ_max_ 206; ESI-MS *m/z*: [M]^+^ calcd. for C_20_H_30_O_3_: 318.22; found, 317.100 ([M]^+^ - [H]^+^, 100%, negative mode).

***In vitro* experiments**

**Table S1.** Percentage inhibition and IC_50_ values of **1-5** against cyclooxygenase enzymes

| **Sample** | **Percentage inhibition at different concentration (%)** | | | | **IC_50_ values**  **(µg/mL)** |
| --- | --- | --- | --- | --- | --- |
|  | **2.5 µg/mL** | **5.0 µg/mL** | **7.5 µg/mL** | **10.0 µg/mL** |  |
| **Cyclooxygenases 1 (COX1) inhibitory assay** | | | | | |
| **1** | 21.82±1.55 | 40.30±3.77 | 47.13±2.71 | 60.44±4.48 | 8.00±0.31 |
| **2** | 16.33±3.00 | 28.31±3.10 | 42.45±1.68 | 50.89±0.60 | 9.75±0.10 |
| **3** | 38.14±0.72 | 61.51±1.84 | 68.34±2.99 | 83.92±2.27 | 3.77±0.06 |
| **4** | 40.81±0.79 | 61.15±0.69 | 72.28±3.77 | 79.55±2.53 | 3.63±0.06 |
| **5** | 42.29±2.05 | 79.53±1.01 | 86.53±1.27 | 92.94±2.46 | 3.01±0.10 |
| **Indomethacin** | 44.41±1.48 | 55.83±1.25 | 78.59±1.28 | 85.87±1.42 | 3.72±0.06 |
| **Cyclooxygenases 2 (COX2) inhibitory assay** | | | | | |
| **1** | 10.71±2.91 | 23.43±3.07 | 42.82±0.72 | 62.85±3.84 | 8.41±0.14 |
| **2** | 34.45±2.11 | 59.16±0.77 | 77.42±0.90 | 84.98±1.17 | 4.07±0.11 |
| **3** | 48.84±0.96 | 63.17±1.41 | 73.28±1.15 | 82.97±0.44 | 2.71±0.17 |
| **4** | 36.77±2.52 | 55.21±0.43 | 67.06±2.47 | 75.14±2.73 | 4.29±0.07 |
| **5** | 46.61±0.90 | 57.24±1.57 | 69.29±3.66 | 79.41±4.39 | 3.29±0.08 |
| **Indomethacin** | 29.80±1.69 | 53.93±1.15 | 67.09±4.85 | 75.74±3.21 | 4.59±0.12 |

*The experiments were done three times (n=3) and the values were presented as mean ± SD*

**Table S2.** Percentage inhibition and IC_50_ values of **1-5** against 5-lipoxygenase (5-LOX) enzyme

| **Sample** | **Percentage inhibition at different concentration (%)** | | | | **IC_50_ values**  **(µg/mL)** |
| --- | --- | --- | --- | --- | --- |
|  | **2.5 µg/mL** | **5.0 µg/mL** | **7.5 µg/mL** | **10.0 µg/mL** |  |
| **1** | 13.45±0.72 | 25.69±2.74 | 41.14±1.43 | 65.39±0.93 | 8.41±0.12 |
| **2** | 12.46±1.45 | 24.47±1.92 | 40.01±4.41 | 51.17±1.35 | 9.78±0.17 |
| **3** | 34.67±1.47 | 59.29±0.66 | 77.49±0.92 | 85.03±1.05 | 4.06±0.07 |
| **4** | 23.18±2.28 | 44.34±1.68 | 55.35±0.65 | 67.15±2.67 | 5.67±0.19 |
| **5** | 30.03±1.04 | 54.08±1.10 | 67.22±4.61 | 75.84±2.97 | 4.58±0.10 |
| **Diclofenac** | 12.44±1.49 | 23.13±4.16 | 42.76±1.82 | 64.41±0.72 | 8.33±0.11 |

*The experiments were done three times (n=3) and the values were presented as mean ± SD*

***In silico* studies**


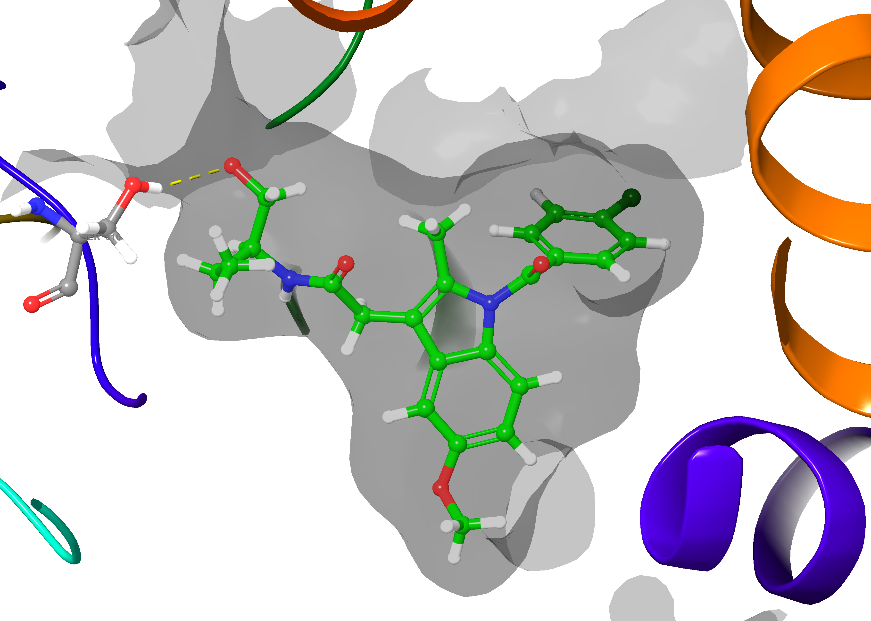

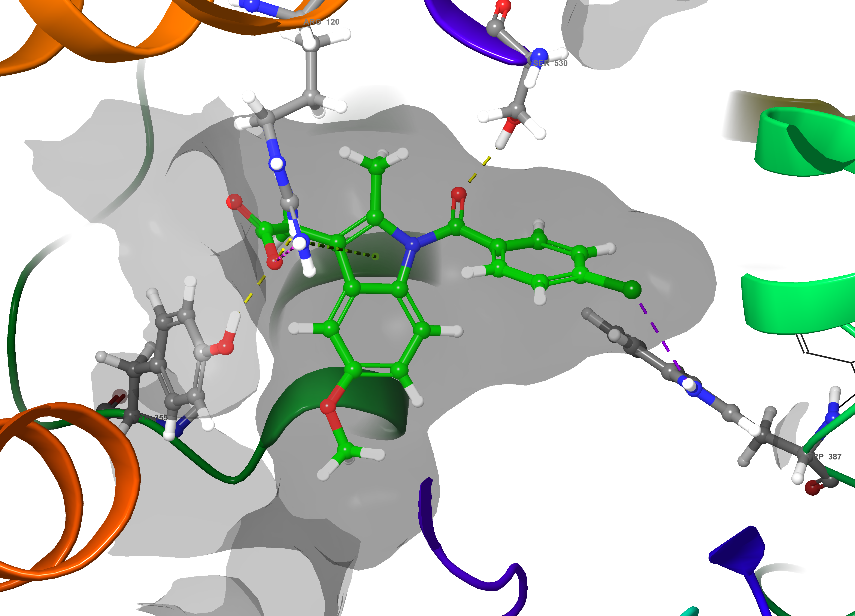


**(a) (b)**

**Figure S16. (a)** Indomethacin-(S)-alpha-ethyl-ethanolamide (green ball-and-stick) bound to COX-1 active site (PDB ID: 2OYU) **(b)** COX-2 complexd with non-selective inhibitor Indomethacin (green ball-and-stick) in crystal structure (PDB ID: 4COX). The grey shape surfaces represent binding region of two proteins.


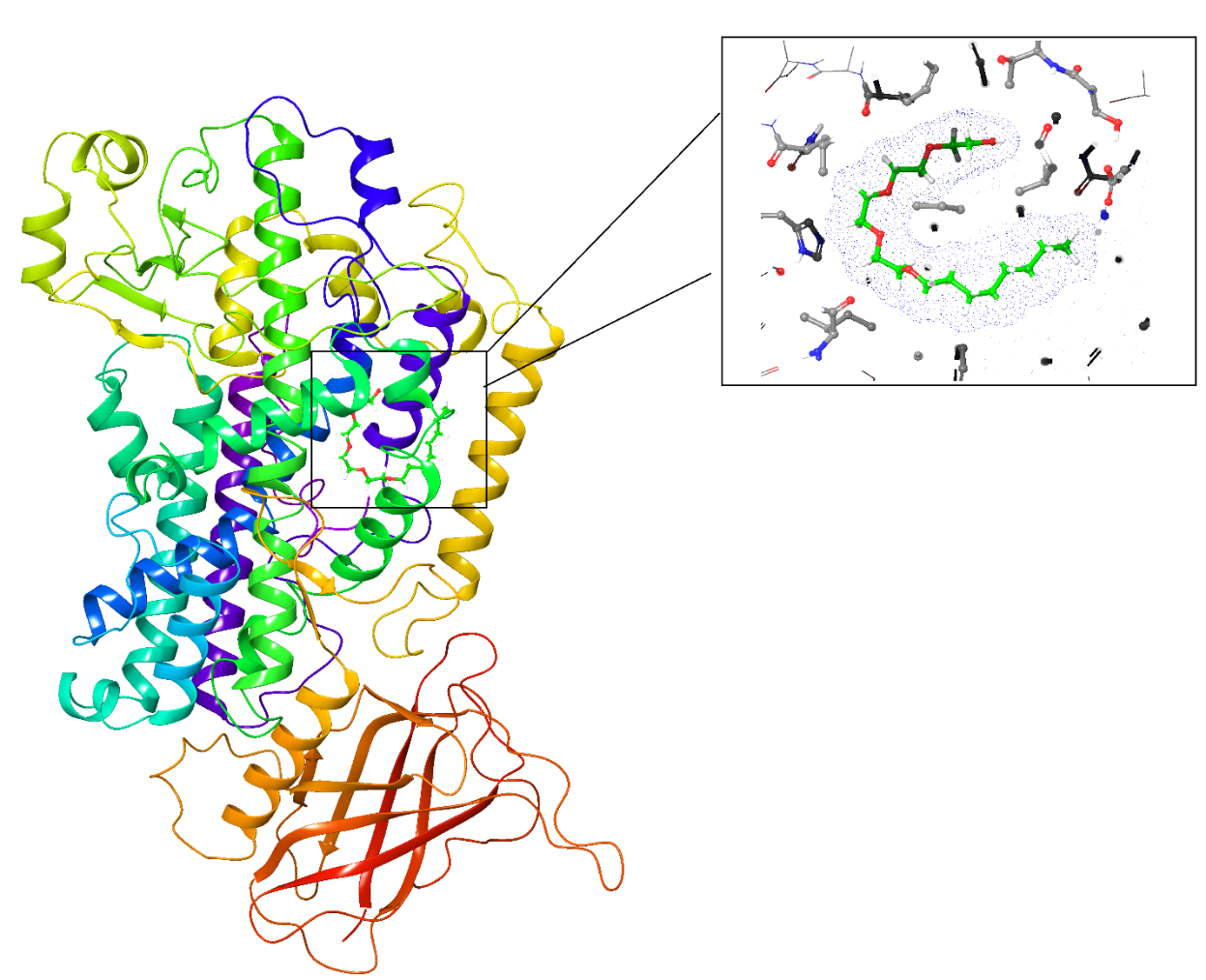
**(a)** **(b)**

**Figure S17.** (**a**) The U-shaped 5-LOX active site (dot-surface) with arachidonate (green wire) in 3V99 crystal structure. (**b**) Biochemical pathway of the conversion of AA into the biologically active leukotrienes. AA is metabolized by 5-LO to 5-hydroperoxyeicosatetraenoic acid (5-HpETE) and leukotriene A4.


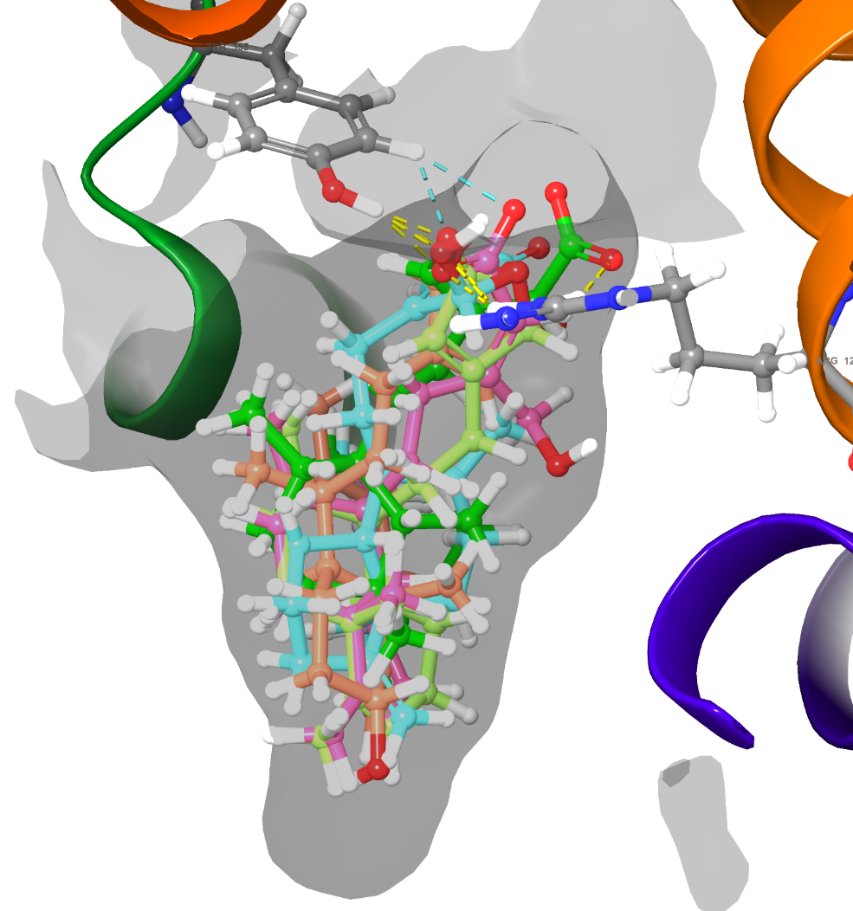


**Figure S18.** The binding poses of all ligands **1**-**5** and two most creating H-bonds residues Tyr355 (grey ball-and-stick left) residues and Arg120 (grey ball-and-stick right). All ligands are well fit in the light grey shape surface of COX-2 binding region


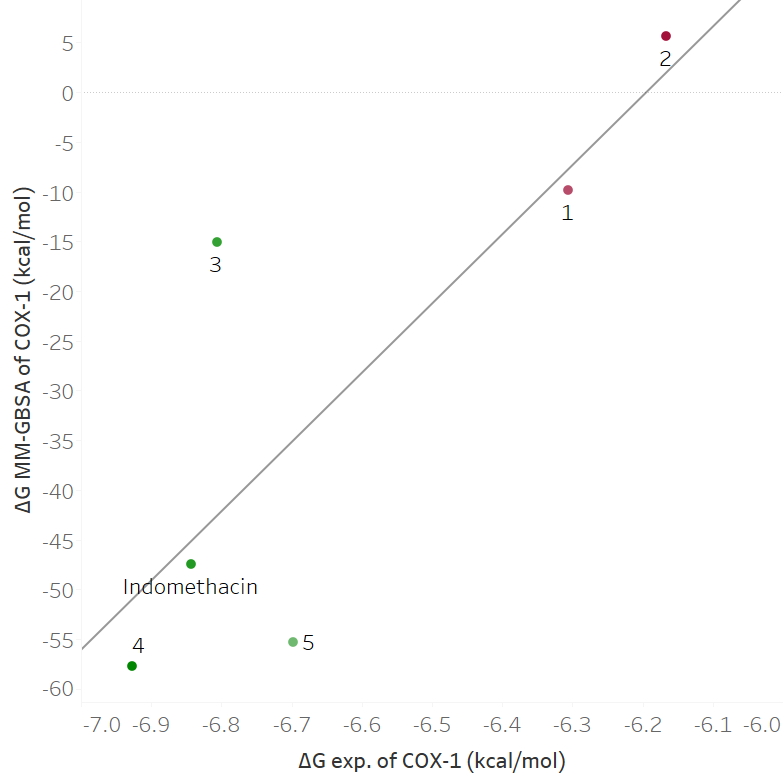


| **P-value:** | 0.05 | | |  | | |
| --- | --- | --- | --- | --- | --- | --- |
| **Equation:** | y = 69.6*x + 431.2 | | |  | | |
| **Coefficients** | | | | | |  |
| **Term** | | **Value** | **StdErr** | | **t-value** | **p-value** |
| Binding energy (COX-1) | | 69.6 | 25.1 | | 2.8 | 0.05 |
| intercept | | 431.2 | 166.8 | | 2.6 | 0.06 |

**Figure S19.** Correlation between
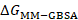
 and
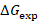
 values of all ligands binding with COX-1 protein. Inhibitor capacity of the ligands is represented by color. The better binding modes have the bolder in green. The darker in red are the weaker inhibitors.


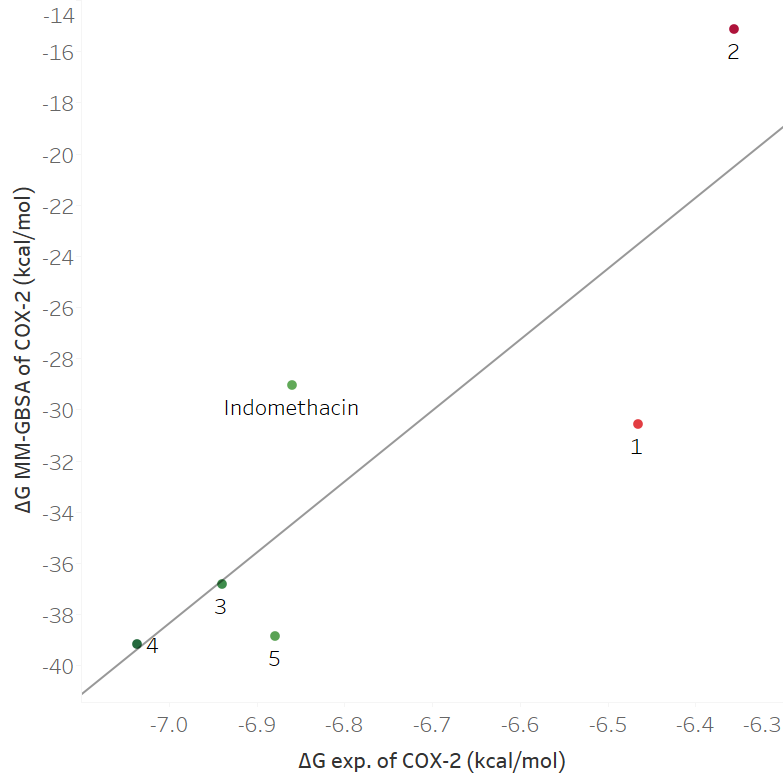


| **P-value:** | 0.04 | | |  | | |
| --- | --- | --- | --- | --- | --- | --- |
| **Equation:** | y = 27.7*x + 155.7 | | |  | | |
| **Coefficients** | | | | | |  |
| **Term** | | **Value** | **StdErr** | | **t-value** | **p-value** |
| Binding energy (COX-2) | | 27.7 | 9.0 | | 3.1 | 0.04 |
| intercept | | 155.7 | 60.5 | | 2.6 | 0.06 |

**Figure S20.** Correlation between
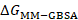
 and
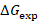
 values of all ligands binding with COX-2 protein. Inhibitor capacity of the ligands is represented by color. The better binding modes have the bolder in green. The darker in red are the weaker inhibitors.


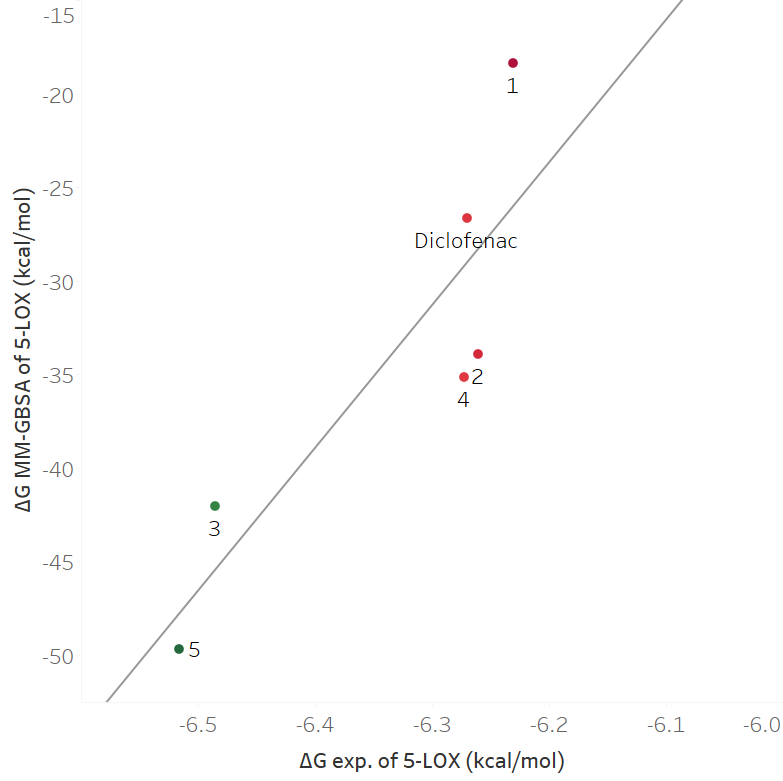


| **P-value:** | 0.02 | | |  | | |
| --- | --- | --- | --- | --- | --- | --- |
| **Equation:** | y = 76.3*x + 449.4 | | |  | | |
| **Coefficients** | | | | | |  |
| **Term** | | **Value** | **StdErr** | | **t-value** | **p-value** |
| Binding energy (5-LOX) | | 76.3 | 21.4 | | 3.6 | 0.02 |
| intercept | | 449.4 | 135.5 | | 3.3 | 0.03 |

**Figure S21.** Correlation between
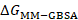
 and
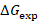
 values of all ligands binding with 5-LOX protein. Inhibitor capacity of the ligands is represented by color. The better binding modes have the bolder in green. The darker in red are the weaker inhibitors.

**References**

1. Greenwood, J. R., Calkins, D., Sullivan, A. P. & Shelley, J. C. Towards the comprehensive, rapid, and accurate prediction of the favorable tautomeric states of drug-like molecules in aqueous solution. *J. Comput. Aided. Mol. Des.* **24**, 591–604 (2010).
2. Shelley, J. C. *et al.* Epik: a software program for pK a prediction and protonation state generation for drug-like molecules. *J. Comput. Aided. Mol. Des.* **21**, 681–691 (2007).
3. Madhavi Sastry, G., Adzhigirey, M., Day, T., Annabhimoju, R. & Sherman, W. Protein and ligand preparation: Parameters, protocols, and influence on virtual screening enrichments. *J. Comput. Aided. Mol. Des.* **27**, 221-234 (2013).
4. Smith, W. L., DeWitt, D. L. & Garavito, R. M. Cyclooxygenases: Structural, cellular, and molecular biology. *Annu. Rev. Biochem.* **69**, 145-182 (2000).
5. Lecomte, M., Laneuville, O., Ji, C., DeWitt, D. L. & Smith, W. L. Acetylation of human prostaglandin endoperoxide synthase-2 (cyclooxygenase-2) by aspirin. *J. Biol. Chem.* **269**, 13207-13215 (1994).
6. Loll, P. J., Picot, D. & Garavito, R. M. The structural basis of aspirin activity inferred from the crystal structure of inactivated prostaglandin H2 synthase. *Nat. Struct. Biol.* **2**, 637-643(1995).
7. Rieke, C. J., Mulichak, A. M., Garavito, R. M. & Smith, W. L. The role of arginine 120 of human prostaglandin endoperoxide H synthase- 2 in the interaction with fatty acid substrates and inhibitors. *J. Biol. Chem.* **274**, 17109-17114 (1999).
8. Saura, P., Maréchal, J.-D., Masgrau, L., Lluch, J. M. & González-Lafont, À. Computational insight into the catalytic implication of head/tail-first orientation of arachidonic acid in human 5-lipoxygenase: consequences for the positional specificity of oxygenation. *Phys. Chem. Chem. Phys.* **18**, 23017–23035 (2016).
9. Gilbert, N. C. *et al.* The structure of human 5-lipoxygenase. *Science.* **331**, 217-219 (2011).
10. Murphy, R. C. & Gijón, M. A. Biosynthesis and metabolism of leukotrienes. *Biochem. J.* **405**, 379-395 (2007).
11. P. Phadnis, A., Patwardhan, S. A., Dhaneshwar, N. N., Tavale, S. S. & Guru Row, T. N. Clerodane diterpenoids from Polyalthia longifolia. *Phytochemistry* **27**, 2899–2901 (1988).
12. Chen, C.-Y. *et al.* Cytotoxic Constituents of Polyalthia longifolia var. p endula. *J. Nat. Prod.* **63**, 1475–1478 (2000).
13. Hara, N. *et al.* Clerodane and ent-halimane diterpenes from polyalthia longifolia. *Phytochemistry* **38**, 189-194 (1995).
14. Sashidhara, K. V., Singh, S. P., Sarkar, J. & Sinha, S. Cytotoxic clerodane diterpenoids from the leaves of Polyalthia longifolia. *Nat. Prod. Res.* **24**, 1687-1694 (2010).
